# Scale validation in applied health research: tutorial for a 6-step R-based psychometrics protocol

**DOI:** 10.1080/21642850.2018.1472602

**Published:** 2018-05-10

**Authors:** Alexandra L. Dima

**Affiliations:** Health Services and Performance Research (HESPER EA 7425), Univ. Lyon, Université Claude Bernard Lyon 1, Lyon, France

**Keywords:** Psychometrics, measurement, item response theory, reproducibility, open science

## Abstract

**Background:** Applied health science research commonly measures concepts via multiple-item tools (scales), such as self-reported questionnaires or observation checklists. They are usually validated in more detail in separate psychometric studies or very cursorily in substantive studies. However, methodologists advise that, as validity is a property of the inferences based on measurement in a context, psychometric analyses should be performed in substantive studies as well. Until recently, performing comprehensive psychometrics required expert knowledge of different, often proprietary, software. The increasing availability of statistical techniques in the R environment now makes it possible to integrate such analyses in applied research.

**Methods:** In this tutorial, I introduce a 6-step protocol which allows detailed diagnosis of core psychometric properties (e.g. structural validity, internal consistency) for scales with binary and ordinal response options aiming to measure differences in degree or quantity, the most common in applied research. The protocol includes investigations of (1) item distributions and summary statistics, item properties via (2) non-parametric and (3) parametric item response theory, (4) scale structure using factor analysis, (5) reliability via classical test theory, and (6) calculation and description of global scores. I illustrate the procedure on a measure of self-reported disability, the 24-item Sickness Impact Profile Roland Scale (RM-SIP), administered in a survey of 222 chronic pain sufferers. An R Markdown script is provided that generates reproducible reports.

**Results:** In this sample, 15 of 24 RM-SIP items formed a unidimensional ordinal scale with good homogeneity (*H* = 0.43) and reliability (*α* = .86[.84–.89]; *ω* = .87[.85–.88]). The two versions were highly correlated (*r* = .96), and regression models predicting RM-SIP disability produced comparable results.

**Conclusions:** The example analysis illustrates how psychometric properties may be assessed in substantive studies and identify avenues for measure improvement. Applied researchers can adapt this script to perform and communicate these analyses as part of questionnaire validation and substantive studies.

When designing a new study, the choice of measurement tools for each of the constructs investigated is a difficult one for applied researchers. In best-case scenarios, there may be appropriate tools available with an already solid validation history based on respondent samples similar to the study population. This becomes more unlikely the more and novel the constructs a study aims to measure, and researchers often have to settle for using measurement tools in contexts for which there is little or no prior psychometric validation, perform measure adaptations within the study, or develop new measures for their specific purposes. For constructs that are not directly observable, multiple-item measures are recommended, such as self-reported questionnaires or observation checklists, which commonly have either binary (e.g. yes/no) or Likert (e.g. strongly agree to strongly disagree) response formats. Thus, psychometric validation of multiple-item tools, or scales, with binary or ordinal formats is an integral part of data analysis in most substantive research studies. This tutorial focuses on scales hypothesized to measure differences in degree or quantity (arguably the most common type of constructs in health research), and proposes a more accessible, comprehensive, and reproducible approach to evaluating core measurement properties as part of substantive research.

Psychometricians highlight the need for ongoing instrument validation, even for established tools, since validity does not reside in the instruments themselves but characterizes the inferences derived from the data generated by the use of an instrument in a given context (Chan, 2014). Yet, despite these recommendations and the sustained progress in psychometric methodology in the last decades, most applied research includes only cursory or no psychometric evaluation of the tools used, which results in the common use of measurement tools with insufficient proof of validity and reliability and raises concerns on the dependability of substantive results (Crutzen & Peters, 2017; Flake, Pek, & Hehman, 2017; Hogan & Agnello, 2004). The benefits of reducing measurement error by improving the psychometric properties of the study questionnaires have been previously illustrated, and include reduction of sample size requirements and study costs (Fries, Krishnan, Rose, Lingala, & Bruce, 2011). In contrast, pervasive measurement error in applied research has sizeable detrimental effects on the accuracy of research findings (Hutcheon, Chiolero, & Hanley, 2010; Marshall et al., 2000), and consequently on the effectiveness of clinical and policy decisions relying on them.

Until recently, the limited availability of data analysis software and training represented a considerable barrier against a more widespread use of psychometric methods in applied research. More advanced methods such as item response theory were available in dedicated software programmes, some proprietary, and only high-resource research groups could afford the time and financial resources to access this expertise. Recent developments in the R environment (R Core Team, 2013) and increased access to online information and instruction have largely overcome this barrier. Numerous R packages on psychometrics methods are now available in the open-source CRAN repository (https://cran.r-project.org). Tutorials describing their practical applications with worked examples with R code are also published with the packages or as methodological articles (Ark, 2007; Dunn, Baguley, & Brunsden, 2014; Mair & Hatzinger, 2007; Rizopoulos, 2007; Stochl, Jones, & Croudace, 2012). Moreover, automatic report generation is possible in R via tools such as Sweave (Leisch, 2002) and R markdown (Allaire, Horner, Marti, & Porte, 2015). Applied researchers have now easier access to using comprehensive and advanced methods for measure validation within substantive studies and reporting the results in a transparent and reproducible way. A key remaining barrier now is time efficiency: even if analyses which used to be time-consuming decades ago now take seconds, a beginning researcher may not afford the time of combining these methods into a streamlined data analysis protocol given the constraints of a typical research project. For such situations, the availability of a general R-based analysis template to adapt to various contexts might help swing the balance in favour of measurement validation within substantive studies.

In this tutorial, I introduce a cross-package psychometrics protocol for researchers with beginner to advanced levels in statistics and R programming interested in investigating core measurement properties (e.g. structural validity, internal consistency) within applied health research projects. I first describe the rationale of including the selected techniques in the protocol, as well as the differences and complementarity between them. I then give an overview of the protocol and provide a worked example of how it can be applied, highlighting the questions answered by each method and how results can be interpreted in the context of an applied study. The corresponding R Markdown script is available as supplementary material, and at https://github.com/alexadima/6-steps-protocol, together with the report generated using this script and the general template researchers may adapt for their own needs (Supplementary Materials 1, 2 and 3). Finally, I discuss its possible uses in applied research in the broader context of generating high-quality evidence for health care. Suggestions for further reading are provided along the way, and summarized in Supplementary Material 4.

## Short journey into measurement theory – or why factor analysis and Cronbach's *α* are not enough

Introductory statistics courses usually describe the process of scale validation as starting with an exploratory factor analysis (EFA), then selecting and interpreting subscales based on the preferred factor solution, and calculating internal consistency per subscale using Cronbach's *α*; based on these results, subscale scores are computed, eventually correlated to other relevant variables to test hypothesized relationships. Although the brevity of these introductory courses is dictated by the time constraints of educational programmes, it may give the erroneous impression that such cursory approach is sufficient; it is not. On the other hand, at advanced levels of expertise in scale development and validation, one may find highly-sophisticated mathematical proofs or comprehensive research lines for establishing measurement properties of widely-used measures (Rabin & Charro, 2001; Skevington, Lotfy, & O’Connell, 2004) or programmes for developing item banks for computer adaptive testing (Cella et al., 2010). Numerous quantitative and qualitative methods are available for assessing various measurement properties. For health-related patient-reported outcomes in evaluative applications, the recent COSMIN consensus taxonomy proposed seven measurement properties in need of testing: face validity, construct validity (structural, cross-cultural, and hypotheses testing), criterion validity, internal consistency, reliability, measurement error, responsiveness (Mokkink et al., 2010). For the applied researcher, psychometrics needs to strike a balance between parsimony and comprehensiveness. Ideally, we would need a standardized yet flexible data analysis protocol to test a core set of measurement properties relevant to most research designs, which can be reproduced for several data and item sets, and modified for study-specific purposes.

The ‘EFA, then Cronbach's *α*’ approach in introductory statistics books rests on the proposal that structural validity and internal consistency represent the core measurement properties requiring proof for most purposes and datasets. The practical advantage of this analysis is that is does not require more data collection (as for other properties such as face validity or criterion validity); the responses to the scale items at one time-point are sufficient to evaluate these properties. The 6-step protocol presented in this tutorial takes this proposal as a starting point, but moves further in ‘psychometrics territory’ based on the following thesis: EFA and Cronbach's *α* are not always the best choice of testing these two properties and may give misleading results, therefore applied researchers need to be conversant in several other techniques in order to perform a meaningful psychometric analysis and avoid erroneous decisions and conclusions. To introduce the alternatives to the basic approach and justify their inclusion in the 6-step protocol, a brief expedition into psychometrics territory is required. Since most readers may be already familiar with basic notions, I will not explain here the reasons for measuring latent constructs via multiple-item tools or the differences between validity (how well a scale measures what it aims to measure) and reliability (to what extent the scores are free from measurement error) (Nunnally & Bernstein, 1994). I will focus rather on highlighting a few landmarks on the theoretical landscape of psychometrics which guided the development of the 6-step protocol.

### Cronbach's *α*

The first landmark is represented by a consistent body of methodological works criticizing the undiscriminating use of Cronbach's *α* as indicator of reliability (term used often interchangeably with internal consistency) in applied research (Cortina, 1993; Dunn et al., 2014; Graham, 2006; Revelle & Zinbarg, 2009; Sijtsma, 2009). I will use here the term reliability to refer to internal consistency reliability; other forms of reliability (inter-rater, test-retest) are also relevant for scale validation (Mokkink et al., 2010) but not included in this protocol. These arguments rest on a deeper critique of the measurement model behind Cronbach's *α*: Classical Test Theory (CTT). In essence, CTT assumes each item is an identical replication of a unique act of measuring a given property; if each act of measurement has an associated random error and error is normally distributed, the more acts one performs the more the errors cancel each other out and the total score becomes a better estimate of the ‘true’ score (Cappelleri, Jason Lundy, & Hays, 2014). In real life, the implausibility of this theoretical assumption quickly becomes obvious, as items cannot be identical replications of each other unless respondents can be completely restored to their state before measurement after answering each item (Borsboom, 2005). In practical terms, Cronbach's *α* is accurate only when items measure the same construct (and thus form a unidimensional scale), and are interchangeable (at least the variances of the true scores of different items are equal) (Dunn et al., 2014). We do not always have good reasons to expect items have these properties, but most importantly we should not take it for granted when we can actually test it. For Cronbach's *α* to be interpretable, we therefore need a confirmation that the items form a unidimensional scale, i.e. test dimensionality. Moreover, it usually provides a ‘lower bound’ of reliability particularly when the scale has fewer items (Sijtsma, 2009). To avoid relying on a single (and potentially biased) estimate, it is recommended to use confidence intervals for Cronbach's *α*, and several other indices of reliability (Crutzen & Peters, 2017; Dunn et al., 2014; Kelley & Cheng, 2012; Revelle & Zinbarg, 2009).

### Factor analysis

The issue of dimensionality leads us to the second psychometric landmark relevant for this protocol: Factor Analysis (FA). Factor models are the most common type of Latent Variable Theory (LVT) models in applied research. LVT represents an alternative measurement model to CTT, which stipulates that the property measured is non-observable and causally determines to different extents the answers given by a respondent to individual items. Numerous LVT models exist, depending on whether latent variables and/or items are conceptualized as categorical or continuous variables (Borsboom, 2008). In factor models commonly-used for scale validation, both latent variables and items are considered as continuous. Hence, a latent variable is a single hypothetical dimension whose ‘existence’ can be inferred from the relationships between the observed items. In practical terms, if items are to be added up or averaged in a total score representing ‘how much’ of a property objects/persons have (i.e. where they are situated on the latent dimension that represents the construct), each item should be sufficiently associated with the other items in the scale. For multi-dimensional constructs, calculating subscale scores is justified if the relationships between items are consistent with the researcher's expectations derived from theory and the process of developing this measure. Factor analysis (FA) is essentially a way of testing whether the observed covariance structure of an item set justifies the use of item scores to calculate (sub)scale scores (structural validity), and comes in two forms: exploratory (EFA), if the structure of the questionnaire is yet unknown and needs to be discovered, or confirmatory (CFA), if hypotheses regarding structure already exist and require testing. In FA, we do not assume that items are ‘clones’ of each other in an abstract world as in CTT, and thus it becomes possible to test dimensionality.

FA has proven useful for many measurement situations, yet the commonly-used models come with the assumptions that items are continuous (interval or quasi-interval scales) and show multivariate normality, which is tenable to the extent that items approach univariate normality (Floyd & Widaman, 1995). In situations where these assumptions do not hold, EFA is likely to group items with similar distributions in separate factors (Schuur, 2003).This may be erroneously interpreted as different dimensions of the construct (instead of an artifact of differences in item distributions), resulting in unfounded and likely less effective clinical or policy decisions. Similarly, violations of the multivariate normality assumption may result in misleading parameter and model fit values in CFA when multiple likelihood (the default option of estimator in most statistics software) is used (Schmitt, 2011). Alternatives have been developed for model estimation (Floyd & Widaman, 1995; Li, 2016), and polychoric or tetrachoric correlation matrices can be used as input in both EFA and CFA to relax these assumptions. While formally some FA models are equivalent to IRT models (Kamata & Bauer, 2008), FA remains limited regarding item-level diagnostics, which constrains interpretation and decisions for scale improvement. Moreover, if FA is performed for item selection and size of factor loadings and (normal) distributions are used to judge item quality, items with low or high difficulty will be excluded, which will result in scales being able to differentiate only between people located at the level of the latent continuum where the other, more similar, items are situated – termed the bandwidth-fidelity problem (Singh, 2004). Assuming a continuous distribution becomes increasingly untenable the fewer the response categories for ordinal items, and particularly for binary items, and alternatives to FA need to be considered.

### Item response theory

The increasingly popular alternative to FA is our third psychometric landmark: item response theory (IRT). IRT is fundamentally based on the same measurement model as FA (LVT), but does not pose assumptions regarding the distributional properties of items. IRT was initially developed for binary items and later expanded for ordinal response formats. For each dichotomy between two response options, IRT estimates a specific probability function of being endorsed depending on item ‘difficulty’ and person ‘ability’ (called item response function; IRF), thus taking into account differences in item distributions. Both items and persons are located on the latent dimension, and the middle point on this dimension represents the location where a person of average ‘ability’ (or ‘intensity’, or ‘amount’ of property) has 50% chance of choosing any option of a dichotomy of average ‘difficulty’ (Hays, Morales, & Reise, 2000).

The most frequently used IRT models assume that latent variables are continuums (interval-level) and model these probabilities as nonlinear monotonic functions, represented graphically as item characteristic curves (ICC; see example in next section). Among IRT models, the Rasch model (for binary items) and the Rating Scale model (for ordinal items) represent the strictest quality standard (Bond & Fox, 2015). The IRFs differ only by item difficulty and have optimal and equal discrimination (i.e. the probability of a of a ‘correct’ response increases in the same way for all items along the latent dimension). If responses of individual persons to individual items fall close to the shape of these IRFs (tested via person and item fit values), each person and item can be represented by a single value on a single dimension, where more able persons are more likely to positively endorse all items, and ‘easier’ items are more likely endorsed by all persons. Hence, respondents with equal total scores are likely to have chosen similar answers to individual items, which means the scores are comparable and can be given a clear interpretation at all levels of the latent continuum (Bond & Fox, 2015). Thus, Rasch models pose in the same time less unnecessary assumptions for non-interval items, and raise the standards for item properties necessary to achieve interval-level measurement for the latent variable. If fit is not reached, several other models can be applied to improve fit to the data by estimating other item parameters (e.g. fixed or variable discrimination, or guessing). The term ‘IRT’ is often used to describe only these less constrained models in contrast to Rasch measurement, due to an essential difference in their objectives: IRT aims to find the best model to explain the data, while Rasch aims to fit the data to the model, i.e. find items with the properties required for valid additive measurement, i.e. adding up two scores is an accurate representation of concatenating (putting together) the quantities measured (Hobart & Cano, 2009). Given that most multiple-item measures are to be used to calculate a total score by summing/averaging item scores, the most relevant for the 6-step model is the Rasch approach, as it asks an essential question for scale validity: are total scores an accurate estimation of the location of the respondent on a latent continuum? For studies in which less constrained IRT models would be more appropriate, several resources are available and R implementations can be added to the protocol (Baker & Kim, 2017; Chalmers, 2012; Mair & Hatzinger, 2007; Rizopoulos, 2007).

IRT offers several advantages to applied researchers. First, accounting for item difficulties results in less biased and more parsimonious estimations of dimensionality compared to FA (Schuur, 2003). Second, since persons and items have their independent locations on this dimension, IRT offers a major advantage compared to FA – the possibility of sample and item-independent measurement, and thus adaptive testing: given a larger item bank, each respondent may have to answer fewer items depending on their responses, resulting in lower respondent burden with equal or improved accuracy (Reise, Ainsworth, & Haviland, 2005). And third, it allows measure developers to maintain the width of the latent continuum (i.e. allow for extreme scores) and diagnose any levels of the latent variable less covered by items (where, by consequence, the test is not able to differentiate well between respondents). This advantage is largely overlooked in measure development and has a surreptitious influence on research results, particularly in intervention studies: weak (or non-significant) effects may also represent a false negative finding (type II error) due to using outcome measures less able to differentiate among the full spectrum of respondents except across a narrow range and thus less sensitive to change (which moves respondents beyond this narrow range found in FA studies). Careful consideration of the full range of the latent variable may prevent type II errors with costly clinical or policy consequences (Fok & Henry, 2015).

### Mokken scaling

Parametric IRT models share with CTT and FA a strong assumption: that latent variables are continuous. This may not be necessary for many constructs in applied research and assessment, as decisions may only require an ordering of respondents, therefore differences in degree, not in quantity (Sijtsma & Molenaar, 2002). IRT also requires relatively large samples of respondents and items to arrive at accurate estimates, particularly for ordinal items, and when sampling does not represent proportionally all levels of the latent continuum (Chen et al., 2014). For item sets measuring ordinal latent variables, or when fewer items or persons are available for measurement, fit with parametric IRT may not be a realistic goal. These considerations take us to our fourth landmark, non-parametric IRT (NIRT), which was developed to address these limitations and has been recommended as preliminary analysis to IRT models (Meijer & Baneke, 2004).

Among the NIRT models, Mokken Scaling Analysis (MSA) represents the ordinal-level version of Rasch and Rating Scale analyses (Schuur, 2003), is available in R in the *mokken* package (Ark, 2007), and has been increasingly applied to health research (Stochl et al., 2012; Watson et al., 2012). According to MSA, a set of items can be used to measure differences in degree between persons/objects if they have three properties: (1) unidimensionality, (2) local independence, and (3) monotonicity. In essence, these mean that (1) endorsing more ‘difficult’ items is related to a higher probability of endorsing ‘easier’ items, while the opposite does not apply, (2) items should be related with each other only via the latent variable they measure, and (3) the probability of endorsing an item should not decrease at higher levels of the latent variable. A fourth property, invariant item ordering, is necessary to meet the ordinal equivalent of the Rasch standard: items should keep the same order of difficulty at all levels of the latent variable; this allows comparisons between groups or datasets (Schuur, 2003; Sijtsma & Hemker, 1998). Idiosyncratic response patterns can be also identified as cases with high number of Guttman errors, i.e. instances when a respondent chooses an answer not consistent with the expected overall pattern, for example not endorsing an easier item while endorsing a more difficult one (Meijer, Niessen, & Tendeiro, 2016). An Automatic Item Selection Procedure (*aisp*) can be used to partition the item set into unidimensional scales (Ark, 2007); items that group with each other into scales are described as ‘scalable’. It achieves a similar purpose with exploratory factor analysis but is insensitive to artifacts due to differences in response frequencies between items, which may bias factor analysis results, particularly in dichotomous cases (Sijtsma & Molenaar, 2002). Therefore, MSA provide informative and more appropriate answers on structural validity, as preliminary analysis to parametric models.

### Descriptive statistics

This overview of more advanced psychometric methods may overshadow the importance of basic descriptive statistics and plotting, which should in fact both precede and follow these more advanced analyses. Item-level descriptives are necessary at first to detect out-of-range or missing values, outliers, reverse-coded items and lack of item variation that would inform data preparation decisions. Once item properties and the structure of the latent construct are clarified and scale scoring is performed, examination of score distributions are required to ascertain any ceiling or floor effects and suitability for further planned analyses.

In sum, instead of a basic ‘EFA, then Cronbach's *α*’ approach, which in practice is often limited to ‘only Cronbach's *α*, and ignore result’ (Flake et al., 2017), a more informative analysis needs to include several complementary analyses, interpret them in relation to theory and study context, detail reasons for measurement choices, and report these transparently. In the following section, I describe the order of the analyses and questions they may answer about an item set.

## The 6-step protocol – example analysis

The methodological considerations reviewed in the previous section indicate that CTT, FA and parametric IRT impose relatively strong assumptions on the item set investigated, while MSA allows testing item properties under a less stringent assumption of an ordinal-level latent variable for both binary and ordinal items. It is particularly useful for assessing (uni)dimensionality, which is assumed by CTT and parametric IRT, and on which FA may give biased results. Therefore, the 6-step protocol proposes to use MSA as the first psychometric method for item examination, after data preparation and descriptive statistics at item level. If MSA criteria are met, parametric IRT tests can further investigate whether both item and person fit are maintained when the latent variable is viewed as a continuum while items are modelled as binary or ordinal level. Factor analyses can be used next to explore or confirm the structure, particularly under assumptions of interval or quasi-interval level and multivariate normality of items. For item sets that prove unidimensional and with adequate properties, CTT analyses can then be performed, particularly for evaluating reliability. Finally, scale or subscale scores can be calculated and summarized. Each step answers specific questions about the properties required from an item set to ensure accurate measurement, as described below. Table 1 summarizes the criteria and decision rules.

**Table 1.** Overview of the 6 steps: questions, statistics and criteria for decision.

Step	Question(s)	Statistics	Decision criteria
1. Descriptives	Items with no/little variation?	Frequencies table (ordered) Barplots of response distributions ORD: Descriptives (mean, SD, etc.)	If insufficient variation (for example <5 endorsements for a response option in a binary item, 95% of responses in a single category) → exclude item or merge categories
	Differences between items regarding their distributions?	As above	If yes → possibility that results of IRT and FA might diverge; perform both and compare
	Negative correlations between items?	Tetrachoric (BI) or Spearman (ORD) inter-item correlation matrix & heatplot	If yes → reverse code items with negative correlations
	Are there respondents with unusual response patterns?	Multivariate outliers (Mahalanobis D^2^ and *χ*^2^ test Q-Q plot)	If yes (e.g. D^2^*p* < .001) → consider excluding if valid reasons exist
2. Non-parametric IRT	Do items form a single scale?	Coefficients of homogeneity (item, item pair, scale)	H < .30 → the scale is not homogeneous (or item is not scalable) → consider excluding items after dimensionality checks (below)
	How many, and which respondents have idiosyncratic response patterns?	Guttman errors	> third quartile + 1.5 interquartile range → examine responses and possible reasons (test administration, data entry errors); check influence on results via sensitivity tests
	Is the scale uni- or multi-dimensional?	Automatic item selection algorithm (*aisp*) at increasing homogeneity levels	If unscalable items (value of 0 in *aisp* table at c</=.30) → exclude one by one and repeat *aisp;* If some items ‘take off’ together (shift scale number at the same c value step) → consider the option of subscales (use theory input)
	Are items associated only via the latent dimension?	Conditional association (local independence test)	if significant violations identified → exclude one by one starting with those with crit values >80 (Schuur, 2003) and repeat.
	Is the probability of endorsing a ‘correct’ response option increasing with increasing levels of the latent dimension?	Monotonicity per subscale	if significant violations identified → exclude one by one starting with those with crit values >80 (Schuur, 2003) and repeat.
	Is the ‘difficulty/intensity’ order of the items the same (invariant) at all levels of the latent dimension?	Invariant item ordering per subscale (ORD: method=‘MIIO’)	if significant violations identified → exclude one by one starting with those with crit values >80 (Schuur, 2003) and repeat, OR consider theory input and purpose of the scale (group comparison needed? item hierarchy needed?)
3. Parametric IRT	Do items form a scale that satisfies requirements of additive measurement?	Rasch (BI) or Rating Scale model (ORD) item fit (infit and outfit); pathway map	If item fit outside the mean squares range of 0.6–1.4 and standardized fit statistics outside +/−2.0 → exclude items one by one
	What is the order of item difficulty? Are there levels of the latent continuum with too many/few items?	Item difficulty estimates; joint ICCs plot;	If items are not ordered according to expectations (if a priori hypotheses exist) → consider excluding items If there item set is too easy/difficult for the sample → consider adding items and/or sampling respondents in the deficient area
	Are item associations explained only by the latent dimension?	2- and 3-way residuals (local independence test)	If significant (*χ*^2^ residuals > 3.5) → consider excluding items involved in several significant residuals
	How many, and which respondents have response patterns that do not fit the model?	person fit	persons with mean squares outside range of 0.6–1.4 and standardized fit statistics outside +/−2.0 → examine responses and possible reasons (test administration, data entry errors); check influence on results via sensitivity tests
	How well is the scale able to differentiate between respondents regarding their ability levels?	Separation reliability; person separation	Reliability <.80, person separation <2 (depending on sample size and use of the scale) → consider adding items or sampling respondents with extreme levels
	Does their difficulty match the ability of the sample?	Person-item map	If too many/few items in some areas of the latent continuum (item saturation/deficiency) → consider excluding items / generating items for further study
4. FA	What is the optimal number of dimensions?	(BI: tetrachoric matrix; ORD: default or polychoric matrix) parallel analysis (PA); Very Simple Structure (VSS); Item cluster analysis (ICLUST)	If the PA, VSS and ICLUST solutions differ → consider examining item level diagnostics and Step 2 and 3 results to explain inconsistency
	Are the data consistent with the hypothesized scale structure?	Confirmatory FA: Model fit (*χ*^2^, CFI, TLI, RMSEA); residuals, parameter estimates (factor loadings, covariances between factors)	Model misfit (TLI ≤ 0.95; CFI ≤ 0.95; RMSEA ≥ 0.06; *χ*^2^ p value ≤ .05); parameter estimates different from hypothesized values → consider examining item level diagnostics and Step 2 and 3 results to explain misfit; consider model improvement based on modification indices
5. CTT	Is the (sub)scale reliable?	*α*, *β*, *ω*, G6. ((BI: tetrachoric matrix; ORD: default or polychoric)	Reliability <.80 or .70 (arbitrary thresholds, interpret with care) → consider adding items (depending on the purposes of the scale and results of Step 2 and 3, e.g. person-item map)
	Are items associated with the total score? Would their exclusion improve reliability?	Item-total associations; Cronbach's *α* if item excluded	Item-total associations <.30, *α* increases if item excluded → consider excluding items (depending on results of Step 2 and 3)
6. Total scores	Do total scores show the expected distribution? Any ceiling/floor effects?	Frequencies, descriptives, histograms, % extreme values	If summary statistics not as expected (e.g. % of respondents with extreme values >15% of the sample) → reconceptualization and/or further scale improvement is necessary

Note: BI: statistics applicable only for binary data; ORD: statistics applicable only for ordinal data; IRT: item response theory; FA: factor analysis; CTT: classical test theory;

### Dataset and script

The use of the 6-step protocol is illustrated with an analysis of a 24-item measure of self-rated disability, the Sickness Impact Profile – Roland Scale (RM-SIP), commonly used in pain conditions (Jensen, Strom, Turner, & Romano, 1992; Roland & Morris, 1983; Stroud, McKnight, & Jensen, 2004). The items refer to various difficulties experienced in daily activities due to pain. The respondents are asked to report whether the items describe their condition over the past few days by ticking a box if they experienced those difficulties. Thus, the items have a binary (yes/no) response format. The scale is hypothesized as unidimensional and the total score sums up the affirmative answers. The dataset includes responses from 222 adults participating in a survey on living with chronic pain conducted in 2007–2008 in the Lothian region, United Kingdom (ethical approval from the NHS Lothian Research Ethics Committee). The dataset and the survey section with the RM-SIP questions are available as Supplementary Materials 5 and 6. To illustrate sensitivity analyses, several other relevant variables were included: background characteristics, the Short-Form McGill Pain Questionnaire (SF-MPQ; Melzack, 1987), and the Brief Illness Perceptions Questionnaire (BIPQ; Broadbent, Petrie, Main, & Weinman, 2006).

The 6-step protocol template and its adaptation to this example analysis (SM1 and SM2) have the same structure, which alternates sections of Markdown text with sections of R code. They produce data analysis reports with an initial summary text, some preparatory code, then progressing through all 6 steps following the same structure: outlining the aims, performing the analysis, displaying results in tables and figures, interpretation and decisions. Commented text signposts the different sections, explains the relevant code, gives advice on analysis choices and criteria for interpretation, and suggests other methods that can be added to the script. For this tutorial, I will assume the readers are familiar with R and R studio and have already conducted statistical analysis in this environment. Many resources are available for familiarization with R and R studio, which include preparing the dataset and setting up your R studio session (Gandrud, 2013; Torfs & Brauer, 2014).

To reproduce the analysis I summarize below, the Scale_validation_-_RM-SIP_analysis_-_SM2.Rmd file (SM2) and the dataset (ChronicPainSurvey.csv, SM5) need to be in the same folder on your computer. Open the Rmd file in RStudio, set your working directory to the source file location and click the ‘Knit to HTML’ option on the upper band of the .Rmd tab. You should now have a .html file identical to SM3 in your folder, as well as some tables and figures in separate files, which can be used for example for a journal publication. I highlight here several elements in each step and illustrate how results can be interpreted based on the example dataset.

### Data preparation

The first section of R code installs and imports the R packages necessary for the analysis, sets up several additional options for markdown, and defines some functions for the analyses performed in this script. The second and third section import the dataset, select the item set for the analysis, check response frequencies, and make any necessary adjustments. In our dataset, it was necessary to transform the response options ‘yes’/’no’ into numeric (1/0). When adapting this script for a new dataset, here you would have the chance to spot any out-of-range or missing values and correct any data entry errors. The next analyses require a dataset of numeric variables with no missing values.

### Step 1: descriptive statistics

Several basic outputs in this step allow familiarization with the data. First, response frequencies for all items, in table and figure format, show whether items have sufficient variation to differentiate respondents. If insufficient variation is identified (e.g. <5 endorsements of a response category in a binary item, 95% of responses in a single category of an ordinal item), the item may need to be excluded from further analyses or response categories merged into fewer categories. Moreover, if there are differences between items in response frequencies this may suggest differences in item intensity/difficulty which can be further explored in Step 2. Second, inter-item correlations are plotted for a first visual diagnosis of items and scale structure. Higher correlations between items of the same subscale can be already visible in the correlation matrix. Negative associations between items may indicate the need to reverse-code items, while items with consistent weak associations with other items may prove to be unscalable in later steps. While this is not necessary for some analyses (such as factor analysis), Step 2 and 3 require all items to be coded in the same direction, e.g from low to high ability. Third, identification of multivariate outliers may identify respondents with idiosyncratic response patterns which may need to be excluded if due to data collection errors.

**Figure 1.**
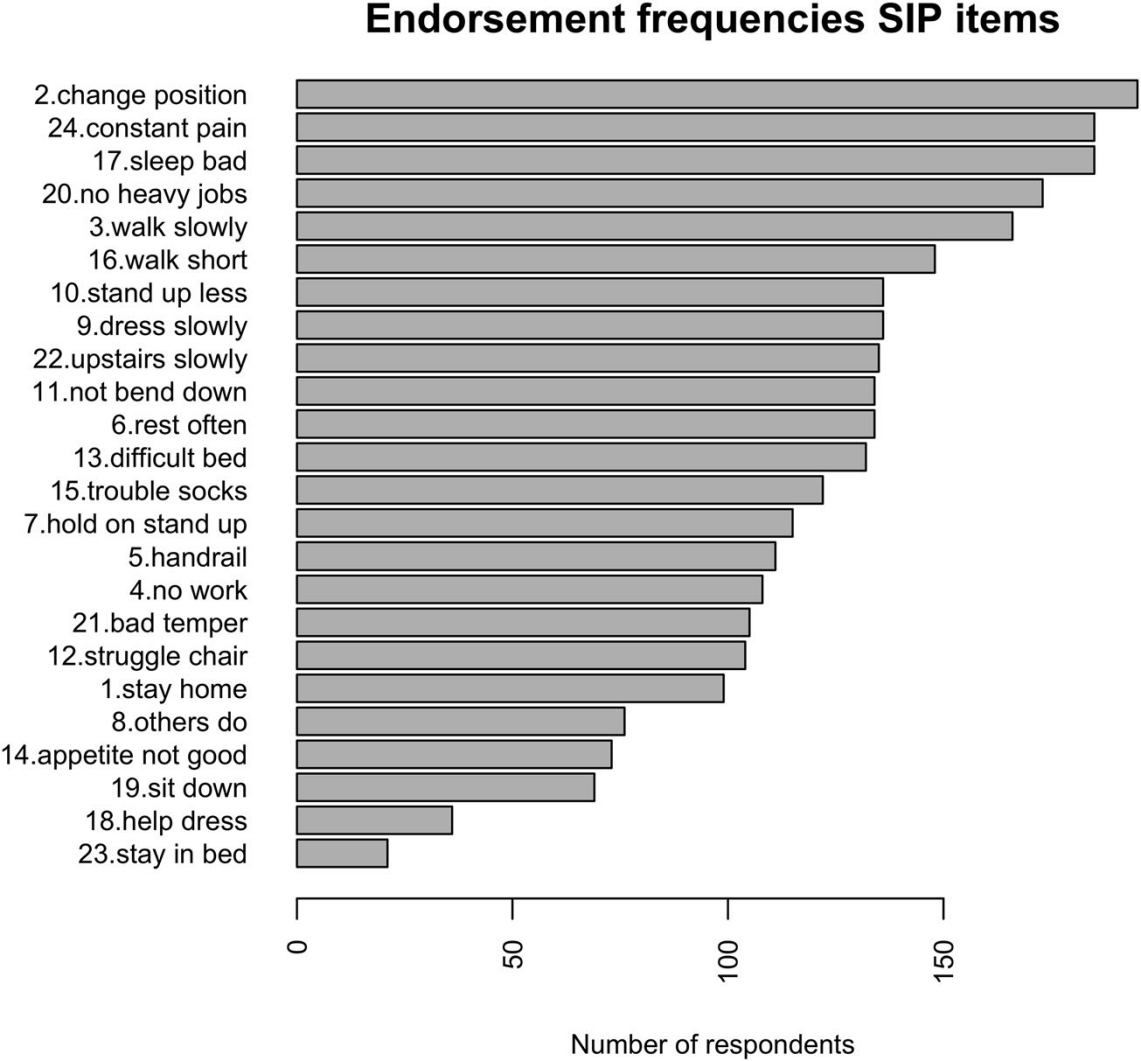
Endorsement frequencies for RM-SIP items from least endorsed to most endorsed.

For the RM-SIP items, this step showed no distribution problems and no negative associations between items, therefore all items were kept in further analyses. No multivariate outliers were found. Figure 1 shows the frequencies of endorsement for all items, in descending order from least endorsed to most endorsed. It can be noted that, while most respondents change position frequently because of their pain, very few stay in bed most of the time. This already suggests that items differ regarding the extent to which they capture the severity of the respondents’ situations. These differences in endorsement frequencies may reflect a latent construct of disability, if they also meet the criteria tested in the next step.

### Step 2: non-parametric item response theory (IRT)

The two item response theory models are applied in steps 2 and 3 to examine item properties for optimal ordinal and interval-level measurement. For non-parametric IRT, the *mokken* package (Ark, 2007) examines coefficients of homogeneity (H) for items, item pairs and scale, and person fit (Guttman errors), and explores possible dimensionality solutions via the *aisp* algorithm performed at increasing thresholds (Hemker, Sijtsma, & Molenaar, 1995). Unscalable items may need to be excluded from further analyses. High numbers of Guttman errors in individual cases may point to data collection errors that require exclusion of cases, and are therefore useful to check. Unidimensional scales identified via *aisp* are subsequently tested for local independence, monotonicity, and invariant item ordering; as explained in the theoretical overview section, item sets that fit these criteria can be considered as measuring a single construct and ordinal differences between respondents are appropriately represented by sum/average scores.Items that show violations of these criteria may be excluded individually, and analyses repeated with the remaining items until an item subset with good performance is found.

For our RM-SIP data, the analyses showed several problematic items. In MSA, the 24-item scale had a homogeneity of *H* = .37(SD = .03), but several items were below the .30 threshold, suggesting they might not measure the same construct. One case was found with a number of Guttman errors higher than the set threshold. The *aisp* analysis (Table 2) identified 3 non-scalable items at a threshold of .30: people may lie down to rest, have no appetite, or be irritable for other reasons rather than the extent of their disability. These items were excluded from further MSA analyses. Six more items did not meet the local independence criterion, and were excluded. The 15-item SIP met the monotonicity and invariant item ordering criteria.

**Table 2.** Mokken Scaling for SIP items: aisp algorithm at increasing homogeneity thresholds.

Item	Homogeneity threshold levels											
	.05	.10	.15	.20	.25	.30	.35	.40	.45	.50	.55	.60
1.stay home	1	1	1	1	1	1	1	1	2	3	2	2
2.change position	1	1	1	1	1	1	1	1	1	1	1	1
3.walk slowly	1	1	1	1	1	1	1	1	1	3	3	3
4.no work	1	1	1	1	1	1	2	2	3	4	0	0
5.handrail	1	1	1	1	1	1	1	1	1	5	7	7
6.rest often	1	1	1	1	0	0	0	0	0	2	0	0
7.hold on stand up	1	1	1	1	1	1	1	1	1	1	1	1
8.others do	1	1	1	1	1	1	2	2	3	4	5	5
9.dress slowly	1	1	1	1	1	1	1	1	1	1	6	6
10.stand up less	1	1	1	1	1	1	1	1	2	3	3	0
11.not bend down	1	1	1	1	1	1	1	1	1	1	1	1
12.struggle chair	1	1	1	1	1	1	1	1	1	1	1	1
13.difficult bed	1	1	1	1	1	1	0	0	0	0	4	4
14.appetite not good	1	1	1	1	1	0	0	0	0	0	0	0
15.trouble socks	1	1	1	1	1	1	1	1	1	1	6	6
16.walk short	1	1	1	1	1	1	1	1	2	3	3	3
17.sleep bad	1	1	1	1	1	1	1	1	1	1	4	4
18.help dress	1	1	1	1	1	1	1	1	1	1	1	1
19.sit down	1	1	1	1	1	1	1	1	2	3	3	3
20.no heavy jobs	1	1	1	1	1	1	1	2	3	4	5	5
21.bad temper	1	1	1	1	1	0	2	0	0	0	0	0
22.upstairs slowly	1	1	1	1	1	1	1	1	1	5	7	7
23.stay in bed	1	1	1	1	1	1	1	1	1	2	2	2
24.constant pain	1	1	1	1	1	1	1	1	1	5	2	2

Note: Numbers represent which subscale the item belongs to; 0 indicates the item is unscalable at that homogeneity level.

### Step 3: parametric item response theory (IRT)

Parametric IRT can be used for unidimensional scales to further diagnose fit with the Rasch model (for binary items) or Rating Scale model (for ordinal items). The *eRm* (Mair & Hatzinger, 2007) and *ltm* (Rizopoulos, 2007) packages provide several item-level diagnostics and visualizations. Item infit and outfit indicate the extent to which actual responses fit the logistic function estimated for a particular item, either giving more weight to answers from respondents whose ability levels are closer to that item's difficulty level (infit), or unweighted (outfit) and thus more sensitive to extreme scorers (Bond & Fox, 2015). Items outside acceptable ranges (see Table 1) are considered for exclusion. Difficulty estimates indicate the location on the latent dimension at which the item is best able to differentiate between respondents. Items not ordered according to theory may need to be excluded. ICCs can be plotted individually or jointly to visualize how item difficulties are distributed across the latent continuum. Pathway maps plot item fit on item difficulty to visualize difficulty levels for problematic items. If items do not cover all the latent dimension, developing new items might be necessary. Residuals for item pairs and triplets indicate whether the inter-item associations are explained by the model, as test of local independence (Bartholomew, 1998). If items are involved in significant residuals they may need to be excluded. Person-level diagnostics (person infit and outfit) are also available to identify which, and how many respondents show anomalous response patterns (number and percentage of persons with over- or underfit). These patterns may need to be individually examined, or sensitivity analyses performed with the remaining cases. Separation reliability and person separation reflect the extent to which the scale is able to differentiate between respondents at different levels of the latent dimension. Person-item maps represent a visual check of how well person abilities and item difficulties match, i.e. the items are overall not too easy or difficult for the sample of respondents (scale targeting). New items may need to be generated if the scale does not differentiate sufficiently overall, or for some areas of the latent continuum; or may be excluded if several items have comparable difficulty levels and are thus redundant. These comprehensive diagnostics, albeit difficult to satisfy fully, identify many options for scale improvement which can be pursued in further research.

**Figure 2.**
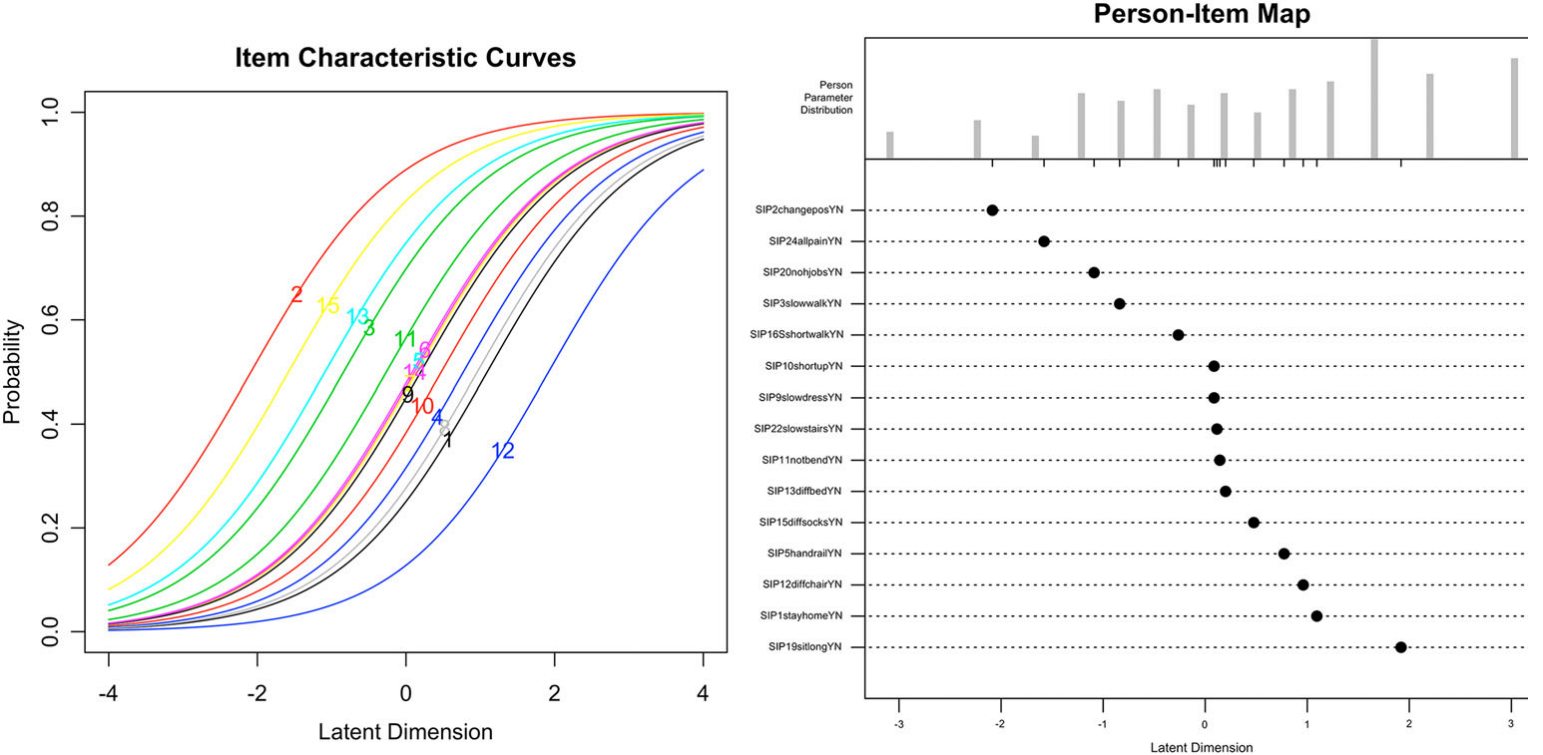
Joint Item Characteristic Curve (ICC) plot and person-item map for the 15-item RM-SIP.

For the RM-SIP data, item and model fit was acceptable in the Rasch analysis. Person reliability and person separation were slightly below the recommended .80 threshold. Several item-pair and item-triplet *χ*^2^ residuals had values > 3.5, indicating local dependencies. These diagnostics suggest that further item exclusion is needed if interval-level (Rasch) measurement is considered necessary, with the drawback of limiting the breadth of the construct. Alternatively, the 15-item RM-SIP could be considered as measuring only an ordinal-level latent variable based on the MSA. This decision would need to consider both theoretical and practical arguments, and is beyond the scope of this tutorial. The latter option was chosen here. Moreover, the joint ICC plot and person-item map (Figure 2) showed item saturation at average levels of the latent variable, and item deficiency at the two extremes. This indicates that, for future scale development, item generation or rewording would need to target the deficient areas while some items with medium difficulty may be excluded.

### Step 4: factor analysis

The *psych* (Revelle, 2017) and *lavaan* (Rosseel, 2012) packages include several factor analysis options for testing scale structure. The interpretation of the number and content of the resulting dimensions may vary depending on the choice of EFA method (Floyd & Widaman, 1995), therefore it is important to consider initially several EFA options, and either compare solutions or select a primary method depending on the context of the study (e.g. replication of published analyses, hypotheses regarding the likely number of dimensions). For ordinal items, the analyses can be performed based on a Pearson or polychoric correlation matrix, while for binary items tetrachoric correlations are appropriate. First, parallel analysis explores the number of factors/components via principal axis factoring or principal component analysis, based on a comparison with simulated/resampled data. It suggests a number of factors/components based on eigenvalues of the real data compared with the mean of the simulated/resampled values. A parallel analysis scree plot displays all values and the number of factors with eigenvalues smaller than the mean eigenvalues of simulated/resampled values. Second, the Very Simple Structure (VSS) analysis determines the optimal number of factors by considering increasing levels of factor complexity (*c*, i.e. the number of factors on which an item loading may differ from zero, up to a pre-specified value). The fit of each factor solution is compared to a simplified loading matrix, in which all except the *c* biggest loadings of each item are set to zero. The VSS plot displays the fit results for each ‘complexity’; the optimal solution is that for which complexity one has the highest value, and thus is easier to interpret. Item cluster analysis (ICLUST) examines similarities between items and generates a bottom-up solution that forms composite scales by grouping items to maximize the value of Cronbach's *α* of the resulting scale, as well as that of the β coefficient, a more conservative estimate of reliability indicating the proportion of variance in the item set accounted for by a general factor by calculating the average covariance between items of the worst split-half, i.e. where separation of the item set in two halves that minimizes this value (Floyd & Widaman, 1995). A cluster graph shows each clustering step and the resulting α and β values; if items cluster together as expected by theory, this can be considered as support for the hypothesized structure. The results of these analyses need to be compared to each other and interpreted in relation to the item-level diagnostics in previous steps. CFA tests a hypothesized structure via model fit statistics and parameter estimates. Model fit indices need to be judged against recommended thresholds: Tucker-Lewis index (TLI) and Comparative Fit Index (CFI) >0.95; root mean square error of approximation (RMSEA) <0.06; and *χ*^2^ p value > .05 (Hu & Bentler, 1999; Jackson, Gillaspy, & Purc-Stephenson, 2009). Good model fit and factor loadings in the expected ranges (as summarized in Table 1) suggest a plausible model. Practical advice for full model diagnosis and reporting is beyond our scope here and can be found in the literature (e.g. Jackson et al., 2009).

**Figure 3.**
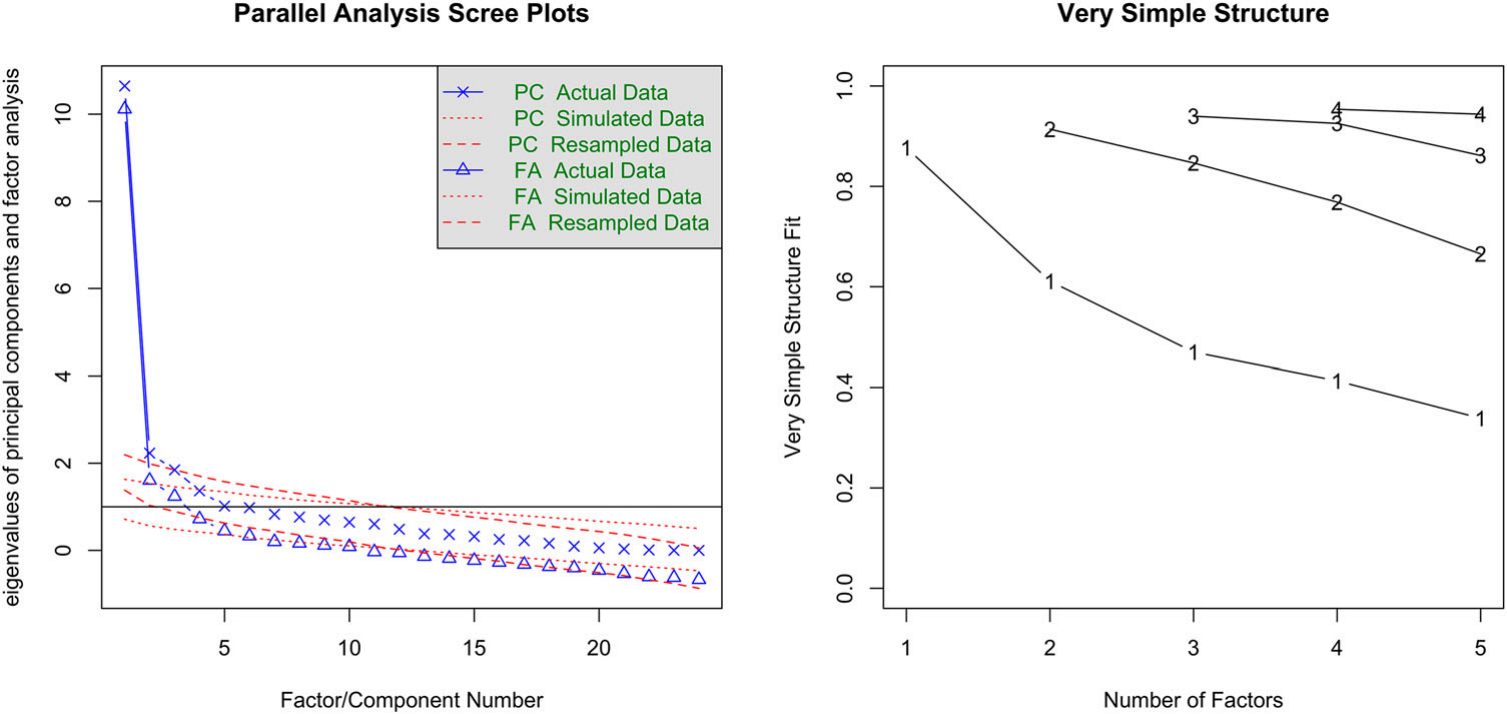
Parallel analysis and Very Simple Structure (VSS) plots for the 24-item RM-SIP.

For the RM-SIP, the parallel analysis based on tetrachoric correlations suggested 6 factors and 3 components, while the VSS was favourable to a unidimensional interpretation (Figure 3). ICLUST concurred with the VSS results. The 1-factor CFAs of the 24-item RM-SIP with the variables specified as ‘ordinal’ showed slightly worse fit (robust *χ*^2^(252)χ 463.99; *p* < .001; CFI = 0.93; TLI = 0.92; RMSEA = 0.06 [0.05–0.07]) compared to the 15-item RM-SIP (robust *χ*^2^(90) = 186.90; *p* < .001; CFI = 0.95; TLI = 0.94; RMSEA = 0.07 [0.06–0.08]), albeit none showed a good fit. Examination of the factor loadings showed that the 3 items unscalable in MSA also had the lowest loadings. Two thresholds commonly considered for item exclusion are <.30 and <.40 (Floyd & Widaman, 1995). In this analysis, the former was not sufficiently sensitive to flag any item, while the latter flagged only two items. This illustrates the importance of using both methods, considering even more conservative thresholds if factor loadings are chosen as criterion for item exclusion, or considering several criteria.

### Step 5: classical test theory

Step 5 calculates reliability (classical test theory) for item (sub)-sets that show unidimensionality via Cronbach's *α* and other reliability indices: *ω*, *β*, and Guttman's lambda 6 (G6) – an alternative estimate based on the item variance accounted for by the linear regression of all remaining items (Revelle & Zinbarg, 2009). Polychoric or tetrachoric correlations may be used for ordinal and binary items, respectively. CTT-based item diagnostics (item-total associations and Cronbach's *α* if item excluded) are also computed. Since steps 4 and 5 represent the basic approach most commonly used in the literature, results are useful for comparison to prior studies, but they are interpretable to the extent that they converge with those of previous steps. Diverging results may suggest invalid assumptions and interpretation needs to link to theory, item content, and data generation process.

For the RM-SIP, reliability indicators between the 24- and 15-item versions did not show substantial differences, and were all within acceptable thresholds. For the 15-item scale, reliability estimates were more consistent with each other, including the β coefficient, which is a more conservative estimate (*α* = .86[.84–.89]; G6 = .87; *β* = .73; *ω* = .87[.85–.88]). The CTT item diagnostics based on reduction in Cronbach's *α* if item excluded were not sensitive enough to identify the 3 unscalable items according to MSA, which illustrates the importance of examining homogeneity, and not only CTT diagnostics, for selecting items that discriminate along a latent continuum.

### Step 6: total (sub)scale scores

Ste 6 computes total scores for unidimensional (sub)scales and calculates summary statistics. For continuous variables, these include ceiling and floor effects, considered acceptable if <15% of respondents have extreme scores (McHorney & Tarlov, 1995). If summary statistics are unsatisfactory, further scale development might be necessary. For the RM-SIP, the total scores for both the 24- and the 15-item versions had acceptable distributions (mean(SD) = 9.2(4.1), range 0–15 and mean(SD) = 9.2(4.1), range 0–15), no ceiling and floor effects, and were highly correlated (*r* = .96).

### Sensitivity analyses

The 6-step analysis identified several items with suboptimal performance. Excluding these items produced a short version that was highly correlated with the original version. To verify the effect that these items had on a substantive question, a sensitivity analysis is included in the script which includes bivariate correlations of the two RM-SIP versions with other variables available in the dataset: 8 items of the BIPQ measuring illness perceptions, and the visual analogue scale of the MPQ measuring pain intensity. Two multiple regression models examined the contribution of illness perceptions, pain intensity, and demographic variables (gender, age, and education) to predicting variance in the two RM-SIP versions. Results were consistent regarding bivariate correlations (Table 3), and the primary role of pain intensity and perceptions of illness consequences in explaining variations in perceived disability (Table 4). There were slight differences concerning perceptions of illness identity and illness concerns, the former showing slightly more substantial role in the original RM-SIP version and the latter in the 15-item version, albeit both estimates were non-significant at a more conservative α level of .01. Thus, substantive conclusions were largely not influenced by the suboptimal performance of some items.

**Table 3.** Bivariate correlations (Pearson's r) between illness perceptions, pain intensity, and 15-item and 24-item RM-SIP.

Variables	IP1	IP2	IP3	IP4	IP5	IP6	IP7	IP8	VAS	SIP24
IP1 – consequences	0.43***									
IP2 – timeline	−0.15*	−0.18**								
IP3 – personal control	−0.15*	−0.15*	0.42***							
IP4 – treatment control	0.53***	0.29***	−0.08	−0.03						
IP5 – identity	0.57***	0.29***	−0.27***	−0.22***	0.41***					
IP6 – concern	0.09	0.06	0.26***	0.30***	0.03	−0.08				
IP7 – understanding	0.50***	0.25***	−0.19**	−0.14*	0.17*	0.60***	−0.11			
Pain intensity – VAS	0.55***	0.30***	−0.22**	−0.15*	0.45***	0.40***	0.12#	0.33***		
24-item RM-SIP	0.64***	0.33***	−0.14*	−0.14*	0.46***	0.35***	0.04	0.35***	0.52***	
15-item RM-SIP	0.61***	0.31***	−0.11	−0.12#	0.44***	0.29***	0.05	0.26***	0.50***	0.96***

Note*:* # *p* < .1; *p < .05; **p < .01; ***p < .001; VAS, visual analogue scale; IP, illness perceptions; RM-SIP, Sickness Impact Profile Roland Scale.

**Table 4.** Multiple regressions of 15-item and 24-item RM-SIP (*n* = 222; non-standardized estimates and standard errors).

	Dependent variable:	
	15-item RM-SIP	24-item RM-SIP
Intercept	−2.241 (1.944)	−3.233 (2.664)
Gender (male)	−0.013 (0.482)	0.320 (0.660)
Age	0.024 (0.020)	0.007 (0.028)
Education (low)	0.805@ (0.454)	0.931 (0.622)
Pain intensity- VAS	0.027** (0.008)	0.034** (0.011)
IP1 – consequences	0.985*** (0.158)	1.285*** (0.216)
IP2 – timeline	0.038 (0.157)	0.112 (0.215)
IP3 – personal control	−0.013 (0.095)	−0.020 (0.131)
IP4 – treatment control	−0.032(0.092)	−0.066 (0.126)
IP5 – identity	0.257@ (0.133)	0.423* (0.183)
IP6 – concern	−0.246* (0.122)	−0.326@(0.167)
IP7 – understanding	−0.028 (0.087)	−0.037 (0.119)
IP8 – emotional response	−0.016(0.117)	0.201 (0.161)
Adjusted R^2^	0.42	0.45

Note*:* ^@^*p* < .1; *p < .05; **p < .01; ***p < .001; VAS, visual analogue scale; IP, illness perceptions; RM-SIP, Sickness Impact Profile Roland Scale.

### Adapting the template

The R Markdown file described above (SM2) has been created by adapting the general template (SM1) to the RM-SIP analysis. A comparison of these documents will help identify the changes required, therefore I will not discuss them individually, but rather highlight the main elements to consider. First, the characteristics of the dataset imported require some obvious adaptations: name and format, location of the items in the dataset, number of items, variable format and labels, scale structure. The template includes advice on how to adapt the code at each step (as commented text), and several options or examples for most common situations. For example, several options for importing data with different formats are given in the data preparation section; while the CFA model in Step 4 would need to be re-written (following the example given in the template for a 3-factor model with 10 items per factor). Second, although most analyses apply to both binary and ordinal items and the same code can be used, some analyses need to be selected depending on the response format. These are indicated in the commented text, as well as in Table 1. And finally, most changes would depend on the results of these analyses and decisions made. For example, some items might need to be excluded if they do not show sufficient variation in Step 1, and do not meet the MSA criteria in Step 2, or the CFA model re-specified if model fit is suboptimal. In such situations, the script of that particular analysis might need to be copied and modified below the initial version and signposted with an explanation of the decisions made and the reasons considered. For interpreting results, the Markdown text in the template provides only some questions to guide interpretation, and obviously needs to be modified by adding the actual interpretation regarding the scale investigated.

## Discussion

This tutorial aimed to demonstrate how applied researchers can acquire in-depth knowledge of scale properties and report their analyses transparently following a 6-step R-based protocol. For the researcher, this allows understanding the concepts, and either confirming scale properties or identifying ways to improve measurement accuracy by excluding items with suboptimal behaviours and performing sensitivity analyses. For the audience (e.g. the wider research team, peer-reviewers, expert users), it allows a better evaluation of measurement quality and its impact on the validity of substantive results.

The example analysis performed on the RM-SIP illustrates the range of information accessible via this protocol. In this sample, the items had variable difficulties, which supports the usefulness of considering this variation in the measurement model. MSA identified several items which did not meet the criteria for ordinal scaling. Yet, 15 of 24 items formed a unidimensional ordinal scale with good homogeneity (*H* = 0.43). Interval scaling requirements were not fully met in Rasch analysis, and scale targeting required improvement. Factor analyses partially converged with IRT findings. The 15-item scale had comparable reliability and distribution with the 24-item scale. The two scores were highly correlated (*r* = .97), and sensitivity analyses for a hypothetical model predicting RM-SIP produced similar results. In sum, in the context of this chronic pain survey, RM-SIP might be considered appropriate to use given the similar results with the 15- and 24-item scale, despite some problematic items. The analysis indicated several ways to improve the scale. If a shorter version would reduce participation burden without loss of information, the 15-item version could be preferred in future research. If the scale would be administered to respondents with more severe disability, it would be necessary to generate more items reflecting higher disability.

The protocol presented here is intended to be used flexibly depending on study purposes and measures investigated. For more elaborate questionnaire development, this protocol may be the start of a more detailed investigation, which may include multidimensional IRT, tests of measurement invariance, stability, responsiveness, etc. The selection of methods and interpretation of results also need to take into account sample size, number and distribution of items and dimensions, measurement context, and hypothesized level of the latent constructs; in this respect, recommendations vary (Anthoine, Moret, Regnault, Sébille, & Hardouin, 2014; Linacre, 1994; MacCallum, Widaman, Zhang, & Hong, 1999; Straat, van der Ark, & Sijtsma, 2014). Providing advice on sample size is beyond the scope of this tutorial. Moreover, since evaluation of psychometric properties is a continuous process of increasing the confidence in a specific tool as well as delimiting its applications for various purposes, this protocol needs to be seen as complementary with examining other sources of validity. Questionnaire development and validation may need to include additional steps besides quantitative analyses, such as conceptual analysis, qualitative research, and adaptation of the tool to different clinical contexts and research designs (Frost, Reeve, Liepa, Stauffer, & Hays, 2007; Peters et al., 2016; Sawatzky et al., 2016). On the other hand, some analyses might be less informative for brief measures, therefore a shorter version of the protocol (e.g. item descriptives, MSA, reliability, and scale scores descriptives) might be sufficient to ensure that calculation of scale scores is justified. Nevertheless, performing all 6 steps in most situations would have relatively low costs and allow checking consistency of results using different methods even in smaller-scale projects.

This protocol is appropriate when the hypothesized latent construct is continuous, and there are several possible conceptualizations that should be considered when developing and validating measures. An alternative way of thinking about differences between persons/objects is in terms of latent categories. In busy clinical settings, grouping people into few easily-identifiable categories may simplify and improve decision making, if categories are clear-cut, relatively stable, and meaningfully related to causes and consequences of the phenomena studied and/or the decisions under consideration. For this type of questions, a more appropriate analysis would be cluster analysis (Clatworthy, Buick, Hankins, Weinman, & Horne, 2005; Everitt, Landau, Leese, & Stahl, 2011; Maechler, Rousseeuw, Struyf, Hubert, & Hornik, 2017), or more sophisticated latent class, latent profile, factor mixture, or grade of membership models (Borsboom et al., 2016). Another possible conceptualization which is increasingly used in mental health starts from the premise that there are no latent characteristics determining responses to individual questions, or the presence or intensity of individual symptoms, but rather items/symptoms influence each other reciprocally to different degrees (Borsboom, 2017). For this approach, network analysis would be more appropriate, and the corresponding statistical functions are implemented in R (Costantini et al., 2015; Epskamp, Borsboom, & Fried, 2017). Careful consideration of the hypothesized nature of the phenomenon under study is essential before choosing to use this protocol or other psychometric methods.

The main scientific benefit of using the 6-step protocol as default scale validation analysis (instead of the basic ‘EFA, then Cronbach's *α*’ approach) is developing the habit of asking fundamental questions about any constructs investigated. Are they intended to capture differences in quantity, or in degree? Do they manage to capture these differences at the level intended? Are they performing equally well between all categories, or at all levels of the ordinal or continuous latent variable? Do all items fit the quality criteria of the model intended or achieved? One may argue that these questions are not a priority for applied researchers, who, ideally, should build on an already well-validated tool box of measures for all purposes and contexts, and focus rather on hypothesis/model testing. In reality, this much-needed tool box is rudimentary, while accurate measurement is increasingly required for both theory testing and real-life decisions. Hence, scale validation often has to be performed by the study team. This protocol aims to facilitate this practical task. For researchers already familiar with R and statistics (including psychometrics), it provides the structure and easy access to different analysis options, which can save time when performing new analyses. For beginners, it provides the key elements and resources for further study; there is however a steep learning curve ahead which should not be underestimated. The analytic choices and interpretations are necessarily left to the user, as they need to be linked to the theory and research objectives.

The coordinated development of theory and measurement has long been considered as best research practice: interpretation of substantive results should consider theoretical statements and measurement issues in equal measure (Nunnally & Bernstein, 1994). Yet, while model testing becomes increasingly sophisticated in substantive studies, progress in measurement methods is comparatively less considered (Hamilton, Marques, & Johnson, 2017). This protocol continues previous efforts to re-balance this state of affairs, such as recent consensus-building initiatives on measurement-related issues (Mokkink et al., 2010; Reeve et al., 2013) and proposals for raising analysis and reporting standards (Crutzen & Peters, 2017). It should be seen as complementary to the ongoing development of PRO databases and item banks, which bring the unquestionable benefit of standardized tools applicable across studies and populations to facilitate evidence synthesis. It can also be taken as a ‘base camp’ for exploring more sophisticated methods, and is intended as a proposal for further debate, and perhaps future consensus on essential criteria for measure validation.

The future of measurement has been presented as technology-mediated adaptive testing relying on large item banks subject to ongoing validation (Cella, Gershon, Lai, & Choi, 2007). These psychometric innovations have the potential to streamline assessment and answer one of the major challenges of moving towards high-functioning ‘learning health systems’ (Friedman et al., 2015). For these innovations to become widely-implemented, sustainable and (cost-)effective in daily practice, it is necessary to move beyond the basics in our research routine. The 6-step protocol presented in this tutorial is a step in this direction.

## Supplementary Material

Scale_validation_-_survey_section_SIP_SM6.doc

ChronicPainSurvey.csv

Scale_validation_-_further_reading_SM4.docx

Scale_validation_-_RM-SIP_analysis_-_SM3.html

Scale_validation_-_RM-SIP_analysis_-_SM2.Rmd

Scale_validation_-_template_analysis_-_SM1.Rmd
