# Supplementary material for: Scale validation in applied health research: tutorial for a 6-step R-based psychometrics protocol
Source: Health Psychol Behav Med. 2018 May 10;6(1):136–61. doi: 10.1080/21642850.2018.1472602 (PMC8133536; doi:10.1080/21642850.2018.1472602)
Supplement: Scale_validation_-_survey_section_SIP_SM6.doc [file RHPB_A_1472602_SM5163.doc]

**GENERAL INFORMATION:**

People adapt in different ways to their health condition, depending on their personality, life experience and habitual ways of dealing with everyday events.

The goal of the present research is to identify what makes your experience of chronic pain unique, in order to help design treatments that reflect individual circumstances.

Your participation in this survey is very important for the ongoing efforts of improving health care services for people with chronic pain.

The present survey intends to measure several aspects of your emotional life and ways of thinking and dealing with your health condition and asks for a few details on your current health status and treatment history.

This study will work best if you attempt to answer all questions. However, if you prefer to omit any question for any reason, please feel free to do so.

Thank you for taking part in this survey!

**[…]**

**ABOUT YOU**

**Before you begin, we would like to ask you some questions about yourself.**

**Please tick the box corresponding to the answer that fits best or fill in the space provided.**

What is your **gender**? 🞎 Male 🞎 Female

What is your **date of birth**? ________ / ________/ ________

Day / Month / Year

What is the highest **education** you received? 🞎 None at all 🞎 Primary school

🞎 Secondary school 🞎 College / University

**[…]**

**This set of questions has been designed to tell us more about your pain. It is important that you tell us how your pain feels now.**

|  | None | Mild | Moderate | Severe |
| --- | --- | --- | --- | --- |
| 1. THROBBING | 🞎 | 🞎 | 🞎 | 🞎 |
| 1. SHOOTING | 🞎 | 🞎 | 🞎 | 🞎 |
| 1. STABBING | 🞎 | 🞎 | 🞎 | 🞎 |
| 1. SHARP | 🞎 | 🞎 | 🞎 | 🞎 |
| 1. CRAMPING | 🞎 | 🞎 | 🞎 | 🞎 |
| 1. GNAWING | 🞎 | 🞎 | 🞎 | 🞎 |
| 1. HOT-BURNING | 🞎 | 🞎 | 🞎 | 🞎 |
| 1. ACHING | 🞎 | 🞎 | 🞎 | 🞎 |
| 1. HEAVY | 🞎 | 🞎 | 🞎 | 🞎 |
| 1. TENDER | 🞎 | 🞎 | 🞎 | 🞎 |
| 1. SPLITTING | 🞎 | 🞎 | 🞎 | 🞎 |
| 1. TIRING-EXHAUSTING | 🞎 | 🞎 | 🞎 | 🞎 |
| 1. SICKENING | 🞎 | 🞎 | 🞎 | 🞎 |
| 1. FEARFUL | 🞎 | 🞎 | 🞎 | 🞎 |
| 1. CRUEL-PUNISHING | 🞎 | 🞎 | 🞎 | 🞎 |

Indicate on this line how bad your pain is - at the left end of line means no pain at all, at right end means worst pain possible.

No Pain _____________________________________________________________________Worst Possible Pain

Which word best describes your pain right now?

| Mild | Discomforting | Distressing | Horrible | Excruciating |
| --- | --- | --- | --- | --- |
| 1 | 2 | 3 | 4 | 5 |

**When you are in pain you may or may not find it difficult to do some of the things you normally do.**

**This list contains some sentences that people have used to describe themselves when they are in pain. When you read them you may find that some stand out because they describe you over the past few days including today.**

**As you read the list, think of yourself. When you read a sentence that describes you put a tick against it. If the sentence does not describe you then leave the space blank and move onto the next one. Remember only to tick the sentence if you are sure that it describes how you have been recently.**

| 1. I stay at home most of the time because of my pain. |  |
| --- | --- |
| 1. I change position frequently to try and get comfortable. |  |
| 1. I walk more slowly than usual because of my pain. |  |
| 1. Because of my pain I am not doing any of the jobs that I usually do around the house. |  |
| 1. Because of my pain I use a handrail to get upstairs. |  |
| 1. Because of my pain I lie down to rest more often. |  |
| 1. Because of my pain I have to hold on to something to get out of an easy chair. |  |
| 1. Because of my pain I try to get other people to do things for me. |  |
| 1. I get dressed more slowly than usual because of my pain. |  |
| 1. I only stand up for short periods of time because of my pain. |  |
| 1. Because of my pain I try not to bend or kneel down. |  |
| 1. I find it difficult to get out of a chair because of my pain. |  |
| 1. I find it difficult to turn over in bed because of my pain. |  |
| 1. My appetite is not very good because of my pain. |  |
| 1. I have trouble putting on my socks (stockings / tights) because of my pain. |  |
| 1. I only walk short distances because of my pain. |  |
| 1. I sleep less well because of my pain. |  |
| 1. Because of my pain I get dressed with help from someone else. |  |
| 1. I sit down for most of the day because of my pain. |  |
| 1. I avoid heavy jobs around the house because of my pain. |  |
| 1. Because of my pain I am more irritable and bad tempered with people than usual. |  |
| 1. Because of my pain I go upstairs more slowly than usual |  |
| 1. I stay in bed most of the time because of my pain. |  |
| 1. I am in pain almost all of the time. |  |

**For the following questions, please circle the number that best corresponds to your views:**

How much does your chronic pain affect your life?

0 1 2 3 4 5 6 7 8 9 10

no affect at all severely affects my life

How long do you think your chronic pain will continue?

0 1 2 3 4 5 6 7 8 9 10

a very short time forever

How much control do you feel you have over your chronic pain?

0 1 2 3 4 5 6 7 8 9 10

absolutely no control extreme amount of control

How much do you think your treatment can help your chronic pain?

0 1 2 3 4 5 6 7 8 9 10

not at all extremely helpful

How much do you experience symptoms from your chronic pain?

0 1 2 3 4 5 6 7 8 9 10

no symptoms at all many severe symptoms

How concerned are you about your chronic pain?

0 1 2 3 4 5 6 7 8 9 10

not at all concerned extremely concerned

How well do you feel you understand your chronic pain?

0 1 2 3 4 5 6 7 8 9 10

don’t understand understand very clearly

at all

How much does your chronic pain affect you emotionally? (e.g. does it make you angry, scared, upset or depressed?)

0 1 2 3 4 5 6 7 8 9 10

not at all affected extremely affected

emotionally emotionally

**Please list in rank-order the three most important factors that you believe caused your chronic pain.**

The most important causes for me:

1. __________________________________

2. __________________________________

3. __________________________________
