# Supplementary material for: Scale validation in applied health research: tutorial for a 6-step R-based psychometrics protocol
Source: Health Psychol Behav Med. 2018 May 10;6(1):136–61. doi: 10.1080/21642850.2018.1472602 (PMC8133536; doi:10.1080/21642850.2018.1472602)
Supplement: Scale_validation_-_further_reading_SM4.docx [file RHPB_A_1472602_SM5161.docx]

Scale validation in applied health research: tutorial for a 6-step R-based psychometric protocol

Supplementary material 4 – selected handbooks:

**Introductions to psychometrics theory:**

Nunnally, J. C., & Bernstein, I. H. (1994). *Psychometric Theory* (3rd ed.). New York, NY US: McGraw-Hill, Inc.

**Item response theory:**

Bond, T., & Fox, C. M. (2015). *Applying the Rasch Model: Fundamental Measurement in the Human Sciences, Third Edition*. Routledge.

**Mokken Scaling:**

Sijtsma, K., & Molenaar, I. W. (2002). *Introduction to Nonparametric Item Response Theory*. SAGE.

**Factor analysis**:

Child, D. (2006). *The Essentials of Factor Analysis*. A&C Black.

**Cluster analysis:**

Everitt, B. S., Landau, S., Leese, M., & Stahl, D. (2011). *Cluster Analysis*. John Wiley & Sons.

**Reproducible research in R:**

Gandrud, C. (2013). *Reproducible Research with R and R Studio*. CRC Press.
