# Supplementary material for: Scale validation in applied health research: tutorial for a 6-step R-based psychometrics protocol
Source: Health Psychol Behav Med. 2018 May 10;6(1):136–61. doi: 10.1080/21642850.2018.1472602 (PMC8133536; doi:10.1080/21642850.2018.1472602)
Supplement: Scale_validation_-_RM-SIP_analysis_-_SM3.html [file RHPB_A_1472602_SM5160.html]

Psychometric analyses 6-steps protocol


# Psychometric analyses 6-steps protocol

### *Example Rmd script*

#### *Author: [name removed for blinded review]; September 2017*

#### *2018-03-03*

# Summary

This document presents the results of a psychometric analysis of the 24-item Sickness Impact Profile Roland Scale (RM-SIP), based on data collected within a survey on living with chronic pain. The RM-SIP has a binary response format (yes/no), is hypothesized as unidimensional and the total score sums up the affirmative answers.

**Step 1** performs basic descriptive statistics at item level.

**Step 2** examines item properties according to non-parametric item response theory (IRT) requirements (Mokken Scaling Analysis; MSA), and parametric (Rasch or Rating Scale Model).

**Step 3** examines item properties according to parametric IRT requirements (Rasch or Rating Scale Model).

**Step 4** examines the structure of the item set according to factor analysis (exploratory and confirmatory).

**Step 5** examines scale reliability and item properties for unidimensional item sets according to Classical Test Theory.

Finally, **step 6** computes total scores and score statistics for each unidimensional item set, and displays distributions as histograms.

The sample size for the dataset analysed is 222.

# Step 1: Descriptives

Response frequencies and item statistics are examined to assess whether items show sufficient variation to be able to differentiate respondents on the construct(s) investigated, and if there are any out-of-range values (data entry errors). Differences in response frequencies also provide a first hint regarding variation in item intensity/difficulty (and help interpret any later differences between IRT and FA results). Associations between items are examined to identify any negative correlations (and reverse code such items for next analyses). Plotting of multivariate outliers helps identify any respondents with idiosyncratic response patterns, which can be further investigated and either excluded (e.g. if errors are identified in the data collection/entry) or kept within the sample (if there are no valid reasons for exclusion).

## Results

The frequencies of endorsing individual response options are presented in Table 1, and barplots of item score distributions are shown in Figure 1.

A heat plot of inter-item correlations (tetrachoric) is shown in Figure 3.

Multivariate outliers in the item set (Mahalanobis distance - D2 values) are displayed graphically in Figure 4.

Table 1: Frequencies of item response options

| Items | YESCount | YESPercentage |
| --- | --- | --- |
| 23.stay in bed | 21 | 9.46% |
| 18.help dress | 36 | 16.22% |
| 19.sit down | 69 | 31.08% |
| 14.appetite not good | 73 | 32.88% |
| 8.others do | 76 | 34.23% |
| 1.stay home | 99 | 44.59% |
| 12.struggle chair | 104 | 46.85% |
| 21.bad temper | 105 | 47.30% |
| 4.no work | 108 | 48.65% |
| 5.handrail | 111 | 50.00% |
| 7.hold on stand up | 115 | 51.80% |
| 15.trouble socks | 122 | 54.95% |
| 13.difficult bed | 132 | 59.46% |
| 6.rest often | 134 | 60.36% |
| 11.not bend down | 134 | 60.36% |
| 22.upstairs slowly | 135 | 60.81% |
| 9.dress slowly | 136 | 61.26% |
| 10.stand up less | 136 | 61.26% |
| 16.walk short | 148 | 66.67% |
| 3.walk slowly | 166 | 74.77% |
| 20.no heavy jobs | 173 | 77.93% |
| 17.sleep bad | 185 | 83.33% |
| 24.constant pain | 185 | 83.33% |
| 2.change position | 195 | 87.84% |

Figure 1: Barplots of high score frequencies

Figure 3: Heatplot Spearman correlations between item scores

Figure 4: Multivariate outliers in item set

There were 0 respondents with D2 values with probability values <.001 (considering a chi-squared distribution with df = the number of items). The maximum D2 value is 44.71.

## Interpretation

No out-of-range values were found. All response options are well-represented in the data (no response category <5 percent) and all associations between items were positive, therefore all items can be included in further analyses. No multivariate outliers were found.

# Step 2: Item properties - Mokken Scaling Analysis (MSA)

Idiosyncratic response patterns are examined within MSA as number of Guttman errors and displayed graphically. Coefficients of homogeneity (H) are examined for the original item set (for each item, item pair, and the overall scale) Values >=.30 indicate scalability.

An Automated Item Selection Procedure (*aisp*) is performed at increasing threshold levels of homogeneity (c) to examine dimensionality. If all items show up as belonging to dimension number 1, this means that the scale is unidimensional at that threshold of homogeneity (indicated in column headings, from .05 to .80). The minimum threshold for homogeneity is .30. Items with a value of 0 are unscalable at that threshold. If at higher threshold levels item separate from dimension number 1 in groups (e.g. 2 or more items ‘leave’ dimension 1 at the same threshold) this indicates that those items may represent a separate dimension. If, on the contrary, items ‘leave’ the dimension one by one and become unscalable, this indicates that there is a single dimension with which items are more or less strongly associated. Unidimensional item subsets are selected based on the *aisp* algorithm (the items selected should show unidimensionality at a threshold level of .30 or higher) and theoretical considerations.

These item subsets are then tested for local independence, monotonicity, and invariant item ordering - 3 criteria for model fit in MSA.

- Local independence is reported as TRUE/FALSE values; if all values are TRUE, the items show local independence with default parameters; if any of the items show up as FALSE, more investigation is needed.
- Monotonicity is shown in table format (default minsize). The ‘zsig’ column shows the number of statistically-significant violations of monotonicity per item; if this number is >0 for one or more items, more investigation of monotonicity is needed (for more testing, various minsize values can be specified in separate tests). Monotonicity is also displayed visually by item step response functions (ISRF; minsize values can be modified to provide a sufficient number of rest score groups for adequate testing).
- invariant item ordering test are shown first in table format (default minsize). The ‘tsig’ column shows the number of statistically-significant violations of IIO per item; if any of the items do not show 0 in the #tsig column, more investigation of IIO is needed (for more testing, various minsize values can be specified in separate tests). IIO is also displayed visually by ISRF plots for each item pair (as above, minsize values can be modified to provide a sufficient number of rest score groups for adequate testing).

For item sets that fit these criteria, it can be concluded that items measure the same construct and total scores can be used to locate respondents on the unidimensional continuum that represent the construct.

## Results

The distribution of Guttman errors is shown in Figure 5.

The homogeneity values of all items in the initial item set are showm in Table 3.

To test unidimensionality, the results of an automated item selection procedure (*aisp*) with all items are shown in Table 4.

Table 5: MSA: Guttman errors for all item set

There were 1 cases with a number of Guttman errors higher than (Q3 plus 1.5 times IQR).

Table 3: MSA: Homogeneity values (and standard errors) for items

| Items | Item H | se |
| --- | --- | --- |
| 1.stay home | 0.425 | (0.041) |
| 2.change position | 0.507 | (0.061) |
| 3.walk slowly | 0.438 | (0.043) |
| 4.no work | 0.330 | (0.043) |
| 5.handrail | 0.369 | (0.038) |
| 6.rest often | 0.213 | (0.045) |
| 7.hold on stand up | 0.399 | (0.037) |
| 8.others do | 0.313 | (0.053) |
| 9.dress slowly | 0.430 | (0.037) |
| 10.stand up less | 0.339 | (0.041) |
| 11.not bend down | 0.405 | (0.038) |
| 12.struggle chair | 0.438 | (0.036) |
| 13.difficult bed | 0.293 | (0.043) |
| 14.appetite not good | 0.241 | (0.057) |
| 15.trouble socks | 0.380 | (0.038) |
| 16.walk short | 0.403 | (0.039) |
| 17.sleep bad | 0.401 | (0.062) |
| 18.help dress | 0.656 | (0.038) |
| 19.sit down | 0.379 | (0.052) |
| 20.no heavy jobs | 0.368 | (0.055) |
| 21.bad temper | 0.271 | (0.046) |
| 22.upstairs slowly | 0.400 | (0.037) |
| 23.stay in bed | 0.526 | (0.087) |
| 24.constant pain | 0.430 | (0.055) |

The complete item set has a homogeneity value H(se) = 0.374, (0.026).

Table 4: MSA: *aisp* for increasing H thresholds (c)

| Items | 0.05 | 0.10 | 0.15 | 0.20 | 0.25 | 0.30 | 0.35 | 0.40 | 0.45 | 0.50 | 0.55 | 0.60 | 0.65 | 0.70 | 0.75 | 0.80 |
| --- | --- | --- | --- | --- | --- | --- | --- | --- | --- | --- | --- | --- | --- | --- | --- | --- |
| 1.stay home | 1 | 1 | 1 | 1 | 1 | 1 | 1 | 1 | 2 | 3 | 2 | 2 | 2 | 2 | 2 | 3 |
| 2.change position | 1 | 1 | 1 | 1 | 1 | 1 | 1 | 1 | 1 | 1 | 1 | 1 | 1 | 1 | 1 | 2 |
| 3.walk slowly | 1 | 1 | 1 | 1 | 1 | 1 | 1 | 1 | 1 | 3 | 3 | 3 | 2 | 0 | 0 | 0 |
| 4.no work | 1 | 1 | 1 | 1 | 1 | 1 | 2 | 2 | 3 | 4 | 0 | 0 | 0 | 0 | 0 | 0 |
| 5.handrail | 1 | 1 | 1 | 1 | 1 | 1 | 1 | 1 | 1 | 5 | 7 | 7 | 0 | 0 | 0 | 0 |
| 6.rest often | 1 | 1 | 1 | 1 | 0 | 0 | 0 | 0 | 0 | 2 | 0 | 0 | 0 | 0 | 0 | 0 |
| 7.hold on stand up | 1 | 1 | 1 | 1 | 1 | 1 | 1 | 1 | 1 | 1 | 1 | 1 | 3 | 0 | 0 | 2 |
| 8.others do | 1 | 1 | 1 | 1 | 1 | 1 | 2 | 2 | 3 | 4 | 5 | 5 | 6 | 6 | 0 | 0 |
| 9.dress slowly | 1 | 1 | 1 | 1 | 1 | 1 | 1 | 1 | 1 | 1 | 6 | 6 | 7 | 0 | 0 | 0 |
| 10.stand up less | 1 | 1 | 1 | 1 | 1 | 1 | 1 | 1 | 2 | 3 | 3 | 0 | 0 | 0 | 0 | 0 |
| 11.not bend down | 1 | 1 | 1 | 1 | 1 | 1 | 1 | 1 | 1 | 1 | 1 | 1 | 1 | 1 | 1 | 1 |
| 12.struggle chair | 1 | 1 | 1 | 1 | 1 | 1 | 1 | 1 | 1 | 1 | 1 | 1 | 3 | 3 | 3 | 4 |
| 13.difficult bed | 1 | 1 | 1 | 1 | 1 | 1 | 0 | 0 | 0 | 0 | 4 | 4 | 5 | 5 | 0 | 0 |
| 14.appetite not good | 1 | 1 | 1 | 1 | 1 | 0 | 0 | 0 | 0 | 0 | 0 | 0 | 0 | 0 | 0 | 0 |
| 15.trouble socks | 1 | 1 | 1 | 1 | 1 | 1 | 1 | 1 | 1 | 1 | 6 | 6 | 7 | 0 | 0 | 0 |
| 16.walk short | 1 | 1 | 1 | 1 | 1 | 1 | 1 | 1 | 2 | 3 | 3 | 3 | 4 | 4 | 0 | 0 |
| 17.sleep bad | 1 | 1 | 1 | 1 | 1 | 1 | 1 | 1 | 1 | 1 | 4 | 4 | 5 | 5 | 0 | 0 |
| 18.help dress | 1 | 1 | 1 | 1 | 1 | 1 | 1 | 1 | 1 | 1 | 1 | 1 | 1 | 1 | 1 | 1 |
| 19.sit down | 1 | 1 | 1 | 1 | 1 | 1 | 1 | 1 | 2 | 3 | 3 | 3 | 4 | 4 | 0 | 0 |
| 20.no heavy jobs | 1 | 1 | 1 | 1 | 1 | 1 | 1 | 2 | 3 | 4 | 5 | 5 | 6 | 6 | 0 | 0 |
| 21.bad temper | 1 | 1 | 1 | 1 | 1 | 0 | 2 | 0 | 0 | 0 | 0 | 0 | 0 | 0 | 0 | 0 |
| 22.upstairs slowly | 1 | 1 | 1 | 1 | 1 | 1 | 1 | 1 | 1 | 5 | 7 | 7 | 0 | 0 | 0 | 0 |
| 23.stay in bed | 1 | 1 | 1 | 1 | 1 | 1 | 1 | 1 | 1 | 2 | 2 | 2 | 2 | 2 | 2 | 3 |
| 24.constant pain | 1 | 1 | 1 | 1 | 1 | 1 | 1 | 1 | 1 | 5 | 2 | 2 | 3 | 3 | 3 | 4 |

**Interpretation**

Based on the aisp table, items 6 14 and 21 are excluded by selecting the remaining items which show unidimensionality at a threshold level of .30. No multi-dimensional solution is apparent from this table (no groups of items identified as ‘leaving to form another scale’ at the same homogeneity threshold).

The 21 items are further examined below for MSA criteria.

The **homogeneity** values of the 18 SIP items are showm in Table 6.

Table 6: MSA: SIP18: item homogeneity values

| ItemS18 | Item H | se |
| --- | --- | --- |
| 1.stay home | 0.437 | (0.044) |
| 2.change position | 0.532 | (0.064) |
| 3.walk slowly | 0.458 | (0.046) |
| 4.no work | 0.334 | (0.047) |
| 5.handrail | 0.408 | (0.040) |
| 7.hold on stand up | 0.452 | (0.038) |
| 8.others do | 0.306 | (0.060) |
| 9.dress slowly | 0.462 | (0.038) |
| 10.stand up less | 0.363 | (0.044) |
| 11.not bend down | 0.441 | (0.039) |
| 12.struggle chair | 0.482 | (0.037) |
| 13.difficult bed | 0.318 | (0.046) |
| 15.trouble socks | 0.410 | (0.041) |
| 16.walk short | 0.436 | (0.041) |
| 17.sleep bad | 0.398 | (0.064) |
| 18.help dress | 0.708 | (0.038) |
| 19.sit down | 0.416 | (0.055) |
| 20.no heavy jobs | 0.386 | (0.057) |
| 22.upstairs slowly | 0.429 | (0.039) |
| 23.stay in bed | 0.509 | (0.100) |
| 24.constant pain | 0.436 | (0.059) |

The 18-item SIP has a homogeneity value H(se) = 0.421, (0.028).

The items flagged as not meeting the **local independence** criterion are presented below :

4.no work, 7.hold on stand up, 8.others do, 17.sleep bad, 18.help dress, 23.stay in bed

These items were excluded from further analysis.

**Monotonicity** tests for the remaining 15 items are shown in Table 7 for default minsize (alternative values of 60 and 50 are displayed below as R output). Item step response functions (minsize=50) are displayed visually in Figure 6

Table 7: MSA: SIP15: monotonicity with default minsize

| Items15 | ItemH | #ac | #vi | #vi/#ac | maxvi | sum | sum/#ac | zmax | #zsig | crit |
| --- | --- | --- | --- | --- | --- | --- | --- | --- | --- | --- |
| 1.stay home | 0.43 | 1 | 0 | 0 | 0 | 0 | 0 | 0 | 0 | 0 |
| 2.change position | 0.5 | 1 | 0 | 0 | 0 | 0 | 0 | 0 | 0 | 0 |
| 3.walk slowly | 0.47 | 1 | 0 | 0 | 0 | 0 | 0 | 0 | 0 | 0 |
| 5.handrail | 0.43 | 1 | 0 | 0 | 0 | 0 | 0 | 0 | 0 | 0 |
| 9.dress slowly | 0.45 | 1 | 0 | 0 | 0 | 0 | 0 | 0 | 0 | 0 |
| 10.stand up less | 0.37 | 1 | 0 | 0 | 0 | 0 | 0 | 0 | 0 | 0 |
| 11.not bend down | 0.43 | 1 | 0 | 0 | 0 | 0 | 0 | 0 | 0 | 0 |
| 12.struggle chair | 0.49 | 1 | 0 | 0 | 0 | 0 | 0 | 0 | 0 | 0 |
| 13.difficult bed | 0.31 | 1 | 0 | 0 | 0 | 0 | 0 | 0 | 0 | 0 |
| 15.trouble socks | 0.41 | 1 | 0 | 0 | 0 | 0 | 0 | 0 | 0 | 0 |
| 16.walk short | 0.45 | 1 | 0 | 0 | 0 | 0 | 0 | 0 | 0 | 0 |
| 19.sit down | 0.44 | 1 | 0 | 0 | 0 | 0 | 0 | 0 | 0 | 0 |
| 20.no heavy jobs | 0.37 | 1 | 0 | 0 | 0 | 0 | 0 | 0 | 0 | 0 |
| 22.upstairs slowly | 0.45 | 1 | 0 | 0 | 0 | 0 | 0 | 0 | 0 | 0 |
| 24.constant pain | 0.43 | 1 | 0 | 0 | 0 | 0 | 0 | 0 | 0 | 0 |

```
##                   ItemH #ac #vi #vi/#ac maxvi sum sum/#ac zmax #zsig crit
## SIP1stayhomeYN     0.43   3   0       0     0   0       0    0     0    0
## SIP2changeposYN    0.50   3   0       0     0   0       0    0     0    0
## SIP3slowwalkYN     0.47   3   0       0     0   0       0    0     0    0
## SIP5handrailYN     0.43   3   0       0     0   0       0    0     0    0
## SIP9slowdressYN    0.45   3   0       0     0   0       0    0     0    0
## SIP10shortupYN     0.37   3   0       0     0   0       0    0     0    0
## SIP11notbendYN     0.43   3   0       0     0   0       0    0     0    0
## SIP12diffchairYN   0.49   3   0       0     0   0       0    0     0    0
## SIP13diffbedYN     0.31   3   0       0     0   0       0    0     0    0
## SIP15diffsocksYN   0.41   3   0       0     0   0       0    0     0    0
## SIP16SshortwalkYN  0.45   3   0       0     0   0       0    0     0    0
## SIP19sitlongYN     0.44   3   0       0     0   0       0    0     0    0
## SIP20nohjobsYN     0.37   3   0       0     0   0       0    0     0    0
## SIP22slowstairsYN  0.45   3   0       0     0   0       0    0     0    0
## SIP24allpainYN     0.43   3   0       0     0   0       0    0     0    0
```

```
##                   ItemH #ac #vi #vi/#ac maxvi sum sum/#ac zmax #zsig crit
## SIP1stayhomeYN     0.43   3   0       0     0   0       0    0     0    0
## SIP2changeposYN    0.50   3   0       0     0   0       0    0     0    0
## SIP3slowwalkYN     0.47   3   0       0     0   0       0    0     0    0
## SIP5handrailYN     0.43   3   0       0     0   0       0    0     0    0
## SIP9slowdressYN    0.45   3   0       0     0   0       0    0     0    0
## SIP10shortupYN     0.37   3   0       0     0   0       0    0     0    0
## SIP11notbendYN     0.43   3   0       0     0   0       0    0     0    0
## SIP12diffchairYN   0.49   3   0       0     0   0       0    0     0    0
## SIP13diffbedYN     0.31   3   0       0     0   0       0    0     0    0
## SIP15diffsocksYN   0.41   3   0       0     0   0       0    0     0    0
## SIP16SshortwalkYN  0.45   6   0       0     0   0       0    0     0    0
## SIP19sitlongYN     0.44   3   0       0     0   0       0    0     0    0
## SIP20nohjobsYN     0.37   3   0       0     0   0       0    0     0    0
## SIP22slowstairsYN  0.45   3   0       0     0   0       0    0     0    0
## SIP24allpainYN     0.43   3   0       0     0   0       0    0     0    0
```

Figure 6: MSA: SIP15: ISRF with minsize=50

**Invariant item ordering (IIO)** tests are shown in Table 8 for default minsize (alternative values of 60 and 50 are displayed below as R output). Intersection plots for tem step response functions (minsize=50) are displayed visually in Figure 7

Table 8: MSA: SIP15: IIO with default minsize

| Items15 | ItemH | #ac | #vi | #vi/#ac | maxvi | sum | sum/#ac | zmax | #zsig | crit |
| --- | --- | --- | --- | --- | --- | --- | --- | --- | --- | --- |
| 1.stay home | 0.43 | 14 | 2 | 0.14 | 0.06 | 0.09 | 0.0066 | 0.87 | 0 | 28 |
| 2.change position | 0.5 | 14 | 0 | 0 | 0 | 0 | 0 | 0 | 0 | 0 |
| 3.walk slowly | 0.47 | 14 | 0 | 0 | 0 | 0 | 0 | 0 | 0 | 0 |
| 5.handrail | 0.43 | 14 | 1 | 0.07 | 0.03 | 0.03 | 0.0024 | 0.38 | 0 | 11 |
| 9.dress slowly | 0.45 | 14 | 3 | 0.21 | 0.1 | 0.2 | 0.0141 | 1.28 | 0 | 50 |
| 10.stand up less | 0.37 | 14 | 2 | 0.14 | 0.06 | 0.13 | 0.009 | 1.25 | 0 | 37 |
| 11.not bend down | 0.43 | 14 | 3 | 0.21 | 0.06 | 0.15 | 0.0104 | 0.77 | 0 | 41 |
| 12.struggle chair | 0.49 | 14 | 1 | 0.07 | 0.06 | 0.06 | 0.0042 | 0.87 | 0 | 15 |
| 13.difficult bed | 0.31 | 14 | 3 | 0.21 | 0.12 | 0.28 | 0.0201 | 1.61 | 0 | 67 |
| 15.trouble socks | 0.41 | 14 | 0 | 0 | 0 | 0 | 0 | 0 | 0 | 0 |
| 16.walk short | 0.45 | 14 | 0 | 0 | 0 | 0 | 0 | 0 | 0 | 0 |
| 19.sit down | 0.44 | 14 | 0 | 0 | 0 | 0 | 0 | 0 | 0 | 0 |
| 20.no heavy jobs | 0.37 | 14 | 0 | 0 | 0 | 0 | 0 | 0 | 0 | 0 |
| 22.upstairs slowly | 0.45 | 14 | 3 | 0.21 | 0.12 | 0.23 | 0.0165 | 1.61 | 0 | 56 |
| 24.constant pain | 0.43 | 14 | 0 | 0 | 0 | 0 | 0 | 0 | 0 | 0 |

```
##                   ItemH #ac #vi #vi/#ac maxvi  sum sum/#ac zmax #zsig crit
## SIP1stayhomeYN     0.43  28   1    0.04  0.04 0.04  0.0015 0.51     0    8
## SIP2changeposYN    0.50  27   0    0.00  0.00 0.00  0.0000 0.00     0    0
## SIP3slowwalkYN     0.47  28   2    0.07  0.06 0.09  0.0031 1.54     0   19
## SIP5handrailYN     0.43  28   2    0.07  0.07 0.10  0.0035 0.74     0   19
## SIP9slowdressYN    0.45  28   4    0.14  0.10 0.33  0.0118 1.50     0   44
## SIP10shortupYN     0.37  28   5    0.18  0.09 0.27  0.0097 1.50     0   47
## SIP11notbendYN     0.43  28   4    0.14  0.10 0.25  0.0088 1.12     0   38
## SIP12diffchairYN   0.49  28   2    0.07  0.07 0.11  0.0038 0.74     0   16
## SIP13diffbedYN     0.31  28   7    0.25  0.16 0.53  0.0190 2.04     1   93
## SIP15diffsocksYN   0.41  28   2    0.07  0.04 0.07  0.0026 0.55     0   15
## SIP16SshortwalkYN  0.45  27   2    0.07  0.05 0.08  0.0029 0.40     0   13
## SIP19sitlongYN     0.44  28   0    0.00  0.00 0.00  0.0000 0.00     0    0
## SIP20nohjobsYN     0.37  28   2    0.07  0.06 0.09  0.0031 1.54     0   24
## SIP22slowstairsYN  0.45  28   5    0.18  0.16 0.42  0.0149 2.04     1   73
## SIP24allpainYN     0.43  28   0    0.00  0.00 0.00  0.0000 0.00     0    0
```

```
##                   ItemH #ac #vi #vi/#ac maxvi  sum sum/#ac zmax #zsig crit
## SIP1stayhomeYN     0.43  29   2    0.07  0.04 0.08  0.0026 0.31     0   12
## SIP2changeposYN    0.50  34   0    0.00  0.00 0.00  0.0000 0.00     0    0
## SIP3slowwalkYN     0.47  32   2    0.06  0.05 0.08  0.0026 0.89     0   14
## SIP5handrailYN     0.43  28   1    0.04  0.03 0.03  0.0012 0.22     0    5
## SIP9slowdressYN    0.45  31   4    0.13  0.10 0.33  0.0107 1.50     0   41
## SIP10shortupYN     0.37  28   5    0.18  0.09 0.27  0.0097 1.50     0   47
## SIP11notbendYN     0.43  31   4    0.13  0.10 0.25  0.0079 1.12     0   36
## SIP12diffchairYN   0.49  30   1    0.03  0.04 0.04  0.0012 0.31     0    3
## SIP13diffbedYN     0.31  29   8    0.28  0.16 0.57  0.0197 2.04     1   96
## SIP15diffsocksYN   0.41  29   3    0.10  0.07 0.14  0.0048 0.71     0   25
## SIP16SshortwalkYN  0.45  31   2    0.06  0.06 0.09  0.0031 1.23     0   19
## SIP19sitlongYN     0.44  35   0    0.00  0.00 0.00  0.0000 0.00     0    0
## SIP20nohjobsYN     0.37  33   3    0.09  0.06 0.15  0.0044 1.23     0   28
## SIP22slowstairsYN  0.45  29   5    0.17  0.16 0.44  0.0151 2.04     1   73
## SIP24allpainYN     0.43  35   0    0.00  0.00 0.00  0.0000 0.00     0    0
```

Table 9: MSA: SIP15: item homogeneity values

| Items15 | Item H | se |
| --- | --- | --- |
| 1.stay home | 0.433 | (0.049) |
| 2.change position | 0.502 | (0.074) |
| 3.walk slowly | 0.473 | (0.047) |
| 5.handrail | 0.432 | (0.042) |
| 9.dress slowly | 0.448 | (0.040) |
| 10.stand up less | 0.370 | (0.046) |
| 11.not bend down | 0.426 | (0.041) |
| 12.struggle chair | 0.487 | (0.040) |
| 13.difficult bed | 0.307 | (0.047) |
| 15.trouble socks | 0.412 | (0.043) |
| 16.walk short | 0.448 | (0.043) |
| 19.sit down | 0.440 | (0.059) |
| 20.no heavy jobs | 0.367 | (0.061) |
| 22.upstairs slowly | 0.454 | (0.038) |
| 24.constant pain | 0.427 | (0.062) |

The 15-item SIP has a homogeneity value H(se) = 0.425, (0.030).

## Interpretation

No idiosyncratic response patterns were found. Three items were not scalable. There were no indications of multidimensionality. All 21 remaining items were scalable at H >= .30. Local independence was not met by 6 items, which were excluded. The remaining 15 items showed monotonicity and invariant item ordering at default rest score group size.

# Step 3: Item properties - parametric Item Response Theory (Rasch Model or Rating Scale Model)

The parametric IRT models includes in this step - the Rasch Model for binary items and Rating Scale Model for ordinal items - follow the same principles with MSA in that they require the probability functions of items in a unidimensional scale to be monotonously increasing, locally independent, and non-intersecting. In contrast to MSA, which tests these properties given an ordinal-level latent, RM and RSM test whether items fit the criteria for additive conjoint measurement, which in essence means that item scores can be added up (or averaged) to a total score that can represent quantitative differences between respondents.

The following diagnostics are reported:

- Item fit (infit and outfit). Criteria for item fit are considered as within the mean squares range of 0.6-1.4 and standardized fit statistics of +/−2.0. If outfit and infit are within these values, they can be considered adequate for measuring the latent construct on an interval-level.
- Global model fit and item-pair and item-triplet residuals (significant results indicate differences between the data and the model, therefore bad fit)
- Item Characteristic Curves are plotted in a joint plot and in separate plots.
- The hierarchy of item difficulty and the match between person ability and item difficulty (scale targeting) are explored graphically via Person-Item map and Pathway map.
- Person reliability (adequate values >.85) and person separation (>2.5) are computed.
- Person fit is evaluated based on the same criteria with item fit (mean squares range of 0.6-1.4 and standardized fit statistics of +/−2.0) and number (and percentages) of misfitting persons are reported based on outfit and infit. (and compared to a criterion of <5 percent)
- the Andersen likelihood ration (LR) test is performed for assessing subgroup homogeneity for high and low scores based on median cut-off.

If (most of) the items in a (sub)scale fit these criteria, it can be concluded that items measure the same construct and total scores can be used to locate respondents on the unidimensional continuum that represent the construct. If fit is not achieved, alternative IRT models may need to be explored to explain the data, or prior MSA results may apply (considering the latent construct at ordinal level).

## Results

**Model fit**, and item-pair and item-triplet residuals for testing **local dependencies** are summarized as output text below.

```
## 
## Goodness-of-Fit Results:
## Collapsed Deviance = 225.136 (df = 210, p-value = 0.225)
## Pearson R2: 0.386
## Area Under ROC: 0.861
```

```
## 
## Information Criteria: 
##                         value npar      AIC      BIC     cAIC
## joint log-lik       -1383.959   28 2823.918 2916.963 2944.963
## marginal log-lik    -1702.613   14 3433.226 3480.864 3494.864
## conditional log-lik -1114.356   14 2256.712 2304.349 2318.349
```

```
## 
## Bootstrap Goodness-of-Fit using Pearson chi-squared
## 
## Call:
## rasch(data = mysubscale2, constraint = cbind(length(mysubscale2) +     1, 1))
## 
## Tobs: 18660.58 
## # data-sets: 200 
## p-value: 0.98
```

```
## 
## Call:
## rasch(data = mysubscale2, constraint = cbind(length(mysubscale2) + 
##     1, 1))
## 
## Fit on the Two-Way Margins
## 
## Response: (0,0)
##   Item i Item j Obs   Exp (O-E)^2/E    
## 1      3     11  40 23.54     11.52 ***
## 2      5     10  70 47.12     11.11 ***
## 3      3     14  44 27.23     10.33 ***
## 
## Response: (1,0)
##   Item i Item j Obs   Exp (O-E)^2/E    
## 1      5     10  30 52.72      9.79 ***
## 2      4     14  16 33.33      9.01 ***
## 3      8     10  20 36.64      7.56 ***
## 
## Response: (0,1)
##   Item i Item j Obs   Exp (O-E)^2/E    
## 1      5     10  16 37.41     12.25 ***
## 2      3     14  12 25.15      6.87 ***
## 3      5      8  16 30.01      6.54 ***
## 
## Response: (1,1)
##   Item i Item j Obs   Exp (O-E)^2/E    
## 1      1     12  53 36.67      7.27 ***
## 2      5     10 106 84.75      5.33 ***
## 3      8     10  84 65.88      4.98 ***
## 
## '***' denotes a chi-squared residual greater than 3.5
```

```
## 
## Call:
## rasch(data = mysubscale2, constraint = cbind(length(mysubscale2) + 
##     1, 1))
## 
## Fit on the Three-Way Margins
## 
## Response: (0,0,0)
##   Item i Item j Item k Obs   Exp (O-E)^2/E    
## 1      3      6     11  37 14.08     37.30 ***
## 2      3     11     14  35 14.20     30.46 ***
## 
## Response: (1,0,0)
##   Item i Item j Item k Obs   Exp (O-E)^2/E    
## 1      5     10     12  15 38.85     14.64 ***
## 2      8     10     12  11 26.25      8.86 ***
## 
## Response: (0,1,0)
##   Item i Item j Item k Obs   Exp (O-E)^2/E    
## 1      1      5     10  11 31.22     13.09 ***
## 2      4      8      9   4 16.37      9.35 ***
## 
## Response: (1,1,0)
##   Item i Item j Item k Obs   Exp (O-E)^2/E    
## 1      4     13     14  11 26.51      9.07 ***
## 2      4      5     14   7 20.26      8.68 ***
## 
## Response: (0,0,1)
##   Item i Item j Item k Obs   Exp (O-E)^2/E    
## 1      5      9     10   4 15.79      8.80 ***
## 2      9     11     14   4 15.75      8.77 ***
## 
## Response: (1,0,1)
##   Item i Item j Item k Obs   Exp (O-E)^2/E    
## 1      6     11     13  11 27.88     10.22 ***
## 2      3      5     10  12 28.21      9.31 ***
## 
## Response: (0,1,1)
##   Item i Item j Item k Obs   Exp (O-E)^2/E    
## 1      5     10     13  11 29.51     11.61 ***
## 2      5     10     15  13 31.69     11.02 ***
## 
## Response: (1,1,1)
##   Item i Item j Item k Obs   Exp (O-E)^2/E    
## 1      5      8     10  76 49.68     13.94 ***
## 2      1     11     12  50 30.45     12.56 ***
## 
## '***' denotes a chi-squared residual greater than 3.5
```

Item characteristic curves are displayed visually in Figure 8, and separately in Figure 13.

The distribution of person latent scores and location of item difficulties on the latent (person-item map) are displayed in Figure 9. Item difficulty and infit statistics are shown in Figure 10.

Figure 8: Rasch: SIP15: Item Characteristic Curves - single plot

Figure 9: Rasch: SIP15: Person-Item Map

Figure 10: Rasch: SIP15: Pathway Map

**Separation reliability** for the 15-item SIP is 0.84, and **person separation** is 2.26.

**Item fit** is shown in Table 10.

Table 10: Rasch: SIP15: Summary item fit

|  | Chisq | df | p-value | Outfit MSQ | Infit MSQ | Outfit t | Infit t |
| --- | --- | --- | --- | --- | --- | --- | --- |
| SIP1stayhomeYN | 199.023 | 204 | 0.585 | 0.971 | 0.977 | -0.14 | -0.26 |
| SIP2changeposYN | 158.742 | 204 | 0.992 | 0.774 | 0.954 | -0.43 | -0.26 |
| SIP3slowwalkYN | 161.866 | 204 | 0.987 | 0.790 | 0.935 | -0.95 | -0.65 |
| SIP5handrailYN | 189.235 | 204 | 0.763 | 0.923 | 0.977 | -0.51 | -0.26 |
| SIP9slowdressYN | 160.308 | 204 | 0.989 | 0.782 | 0.852 | -1.56 | -1.83 |
| SIP10shortupYN | 195.940 | 204 | 0.645 | 0.956 | 1.051 | -0.26 | 0.62 |
| SIP11notbendYN | 156.915 | 204 | 0.994 | 0.765 | 0.911 | -1.73 | -1.07 |
| SIP12diffchairYN | 160.910 | 204 | 0.988 | 0.785 | 0.860 | -1.52 | -1.83 |
| SIP13diffbedYN | 278.781 | 204 | 0.000 | 1.360 | 1.210 | 2.32 | 2.40 |
| SIP15diffsocksYN | 186.567 | 204 | 0.804 | 0.910 | 0.974 | -0.63 | -0.30 |
| SIP16SshortwalkYN | 147.627 | 204 | 0.999 | 0.720 | 0.914 | -1.81 | -0.98 |
| SIP19sitlongYN | 232.402 | 204 | 0.084 | 1.134 | 1.118 | 0.63 | 1.37 |
| SIP20nohjobsYN | 249.493 | 204 | 0.016 | 1.217 | 1.131 | 0.89 | 1.24 |
| SIP22slowstairsYN | 149.772 | 204 | 0.998 | 0.731 | 0.835 | -2.00 | -2.06 |
| SIP24allpainYN | 253.370 | 204 | 0.011 | 1.236 | 1.099 | 0.78 | 0.82 |

Item outfit values ranged between 0.72 and 1.36. Item infit values ranged between 0.84 and 1.21.

There were 0 persons with misfit according to outfit values (representing 0 percent), and 0 persons according to infit values (representing 0 percent of all participants).

Item difficulty estimates (and confidence elipses) for high and low latent score groups are displayed visually in Figure 11

Figure 11: Rasch: SIP15: Item difficulty for high and low latent score groups

Item parameter confidence intervals based on LR test are displayed visually in Figure 12

Figure 12: Rasch: SIP15: Item parameter confidence intervals based on LR test

## Interpretation

All items had acceptable fit (infit and outfit within the recommended range). The global model fit was nonsignificant, but there were a number of significant item-pair and item-triplet residuals indicating possible local dependencies. The person-item map indicated item saturation around average levels of the latent, and item deficiency at the extremes (+/- 2). Items tended to be rather ‘easy’ for these respondents, meaning that respondents with higher disability levels were present and the scale was less able to differentiate between them. Person reliability and person separation were slightly below the recommended thresholds. All persons fitted the model. Many items showed differences in difficulty between subgroups of high and low scores.

Therefore, the 15-item SIP can be considered as not meeting the Rasch criteria. Excluding more items would reduce the content validity of the scale, with little gain in precision. Therefore, the scale can be considered as measuring only an ordinal-level latent (according to MSA).

# Step 4: Factor analysis

Exploratory and confirmatory factor analyses are performed to provide a complementary perspective on the dimensionality of the item set, under various conditions. Unlike item response theory, factor analysis treats items as continuous variables with comparable (normal) distributions; thus, it does not acknowledge differences between items in terms of the probability of response options being endorsed by respondents at different levels of the latent continuum measured (item difficulty/intensity). Therefore, factor analysis can provide different results compared to IRT methods. Results that confirm the structure identified in Step 2 and 3 can be considered as enforcing the findings (i.e. results do not depend on the method used); differences in results may require a reconsideration of these assumptions (e.g. FA can also be run treating items as ordinal by using a polychoric correlation matrix). As IRT analyses are less common, FA analyses can also be used to compare results of a new study with analyses reported in previous studies. The following analyses are performed:

- parallel analysis explores the number of factors/components via principal components and principal axis factoring, based on a comparison with simulated/resampled data. It suggests a number of factors/components based on eigenvalues (default is by comparing them with the mean of the simulated/resampled values). The solution is displayed as R console output text, and graphically as a screeplot.
- Very Simple Structure (VSS) analysis determines the optimal number of factors by considering increasing levels of factor complexity (c, i.e. the number of factors on which an item loading may differ from zero, up to a pre-specified value). The fit of each factor solution is compared to a simplified loading matrix, in which all except the c biggest loadings of each item are set to zero. The VSS plot displays the fit results for each ‘complexity’; the optimal solution is that for which complexity one has the highest value, and thus is easier to interpret. The results are also reported as text output.
- Item cluster analysis (ICLUST) is an alternative to factor analysis that examines the similarities between items and explores a bottom-up solution that forms composite scales by grouping items so that alpha and beta coefficients of the resulting scales increase (the default option is applied here, several parametrizations available). The results are visualized in a cluster graph that shows the steps of clustering and the resulting alpha and beta coefficients; if items cluster together as expected by theory, this can be considered as support for the hypothesized structure. Output text provides reliability indicators for the resulting scale(s).
- Confirmatory factor analysis based on theory reports fit statistics and parameter estimates for a pre-specified model (1-factor solution for SIP). Results are presented in diagram form, and also as output text. Model fit indices above recommended thresholds (Tucker-Lewis index (TLI) and Comparative Fit Index (CFI) >0.95; root mean square error of approximation (RMSEA) <0.06; chi-square P value >.05) and factor loadings in the expected ranges (>.30 or .40 for the hypothesised dimensions) suggest a plausible model.

## Results

```
## Parallel analysis suggests that the number of factors =  6  and the number of components =  3
```

Figure 14: FA: Parallel analysis screeplot & Very Simple Structure plot

```
## 
## Very Simple Structure
## Call: vss(x = mydata, n = 5, cor = "tet")
## VSS complexity 1 achieves a maximimum of 0.88  with  1  factors
## VSS complexity 2 achieves a maximimum of 0.91  with  2  factors
## 
## The Velicer MAP achieves a minimum of 0.04  with  3  factors 
## BIC achieves a minimum of  9412.57  with  5  factors
## Sample Size adjusted BIC achieves a minimum of  9938.63  with  5  factors
## 
## Statistics by number of factors 
##   vss1 vss2   map dof chisq prob sqresid  fit RMSEA   BIC SABIC complex
## 1 0.88 0.00 0.045 252 11600    0    15.9 0.88  0.46 10238 11037     1.0
## 2 0.61 0.91 0.042 229 11183    0    11.1 0.91  0.48  9946 10672     1.4
## 3 0.47 0.85 0.041 207 10814    0     7.8 0.94  0.49  9696 10352     1.7
## 4 0.41 0.77 0.041 186 10519    0     6.0 0.95  0.52  9514 10103     2.0
## 5 0.34 0.67 0.047 166 10309    0     5.1 0.96  0.54  9413  9939     2.3
##   eChisq  SRMR eCRMS eBIC
## 1   1627 0.115 0.121  266
## 2   1006 0.091 0.099 -231
## 3    595 0.070 0.080 -524
## 4    399 0.057 0.070 -606
## 5    314 0.051 0.065 -583
```

Figure 15: FA: hierarchical cluster analysis for items (ICLUST)

Confirmatory factor analysis:

24-item SIP:

```
## lavaan (0.5-23.1097) converged normally after  27 iterations
## 
##   Number of observations                           222
## 
##   Estimator                                       DWLS      Robust
##   Minimum Function Test Statistic              422.136     463.988
##   Degrees of freedom                               252         252
##   P-value (Chi-square)                           0.000       0.000
##   Scaling correction factor                                  1.178
##   Shift parameter                                          105.773
##     for simple second-order correction (Mplus variant)
## 
## Model test baseline model:
## 
##   Minimum Function Test Statistic             6694.860    3166.123
##   Degrees of freedom                               276         276
##   P-value                                        0.000       0.000
## 
## User model versus baseline model:
## 
##   Comparative Fit Index (CFI)                    0.973       0.927
##   Tucker-Lewis Index (TLI)                       0.971       0.920
## 
##   Robust Comparative Fit Index (CFI)                            NA
##   Robust Tucker-Lewis Index (TLI)                               NA
## 
## Root Mean Square Error of Approximation:
## 
##   RMSEA                                          0.055       0.062
##   90 Percent Confidence Interval          0.046  0.064       0.053  0.070
##   P-value RMSEA <= 0.05                          0.171       0.016
## 
##   Robust RMSEA                                                  NA
##   90 Percent Confidence Interval                                NA     NA
## 
## Standardized Root Mean Square Residual:
## 
##   SRMR                                           0.119       0.119
## 
## Weighted Root Mean Square Residual:
## 
##   WRMR                                           1.186       1.186
## 
## Parameter Estimates:
## 
##   Information                                 Expected
##   Standard Errors                           Robust.sem
## 
## Latent Variables:
##                    Estimate  Std.Err  z-value  P(>|z|)   Std.lv  Std.all
##   SIP =~                                                                
##     SIP1stayhomeYN    1.000                               0.733    0.733
##     SIP2changepsYN    0.972    0.117    8.318    0.000    0.712    0.712
##     SIP3slowwalkYN    1.010    0.108    9.365    0.000    0.740    0.740
##     SIP4notdoYN       0.819    0.096    8.487    0.000    0.600    0.600
##     SIP5handrailYN    0.983    0.095   10.326    0.000    0.720    0.720
##     SIP6liedownYN     0.545    0.111    4.893    0.000    0.399    0.399
##     SIP7holdonYN      1.099    0.093   11.790    0.000    0.805    0.805
##     SIP8othersdoYN    0.693    0.109    6.329    0.000    0.508    0.508
##     SIP9slowdrssYN    1.113    0.099   11.247    0.000    0.816    0.816
##     SIP10shortupYN    0.898    0.093    9.665    0.000    0.658    0.658
##     SIP11notbendYN    1.049    0.094   11.125    0.000    0.768    0.768
##     SIP12diffchrYN    1.130    0.095   11.953    0.000    0.828    0.828
##     SIP13diffbedYN    0.794    0.100    7.935    0.000    0.582    0.582
##     SIP14noappetYN    0.513    0.110    4.683    0.000    0.376    0.376
##     SIP15dffscksYN    1.019    0.099   10.322    0.000    0.746    0.746
##     SIP16SshrtwlYN    1.006    0.096   10.502    0.000    0.737    0.737
##     SIP17badslepYN    0.834    0.119    6.994    0.000    0.611    0.611
##     SIP18hlpdrssYN    1.158    0.102   11.335    0.000    0.848    0.848
##     SIP19sitlongYN    0.826    0.098    8.432    0.000    0.605    0.605
##     SIP20nohjobsYN    0.795    0.108    7.360    0.000    0.583    0.583
##     SIP21irritblYN    0.658    0.106    6.221    0.000    0.482    0.482
##     SIP22slwstrsYN    1.046    0.096   10.920    0.000    0.766    0.766
##     SIP23staybedYN    0.779    0.141    5.515    0.000    0.571    0.571
##     SIP24allpainYN    0.872    0.116    7.538    0.000    0.639    0.639
## 
## Intercepts:
##                    Estimate  Std.Err  z-value  P(>|z|)   Std.lv  Std.all
##    .SIP1stayhomeYN    0.000                               0.000    0.000
##    .SIP2changepsYN    0.000                               0.000    0.000
##    .SIP3slowwalkYN    0.000                               0.000    0.000
##    .SIP4notdoYN       0.000                               0.000    0.000
##    .SIP5handrailYN    0.000                               0.000    0.000
##    .SIP6liedownYN     0.000                               0.000    0.000
##    .SIP7holdonYN      0.000                               0.000    0.000
##    .SIP8othersdoYN    0.000                               0.000    0.000
##    .SIP9slowdrssYN    0.000                               0.000    0.000
##    .SIP10shortupYN    0.000                               0.000    0.000
##    .SIP11notbendYN    0.000                               0.000    0.000
##    .SIP12diffchrYN    0.000                               0.000    0.000
##    .SIP13diffbedYN    0.000                               0.000    0.000
##    .SIP14noappetYN    0.000                               0.000    0.000
##    .SIP15dffscksYN    0.000                               0.000    0.000
##    .SIP16SshrtwlYN    0.000                               0.000    0.000
##    .SIP17badslepYN    0.000                               0.000    0.000
##    .SIP18hlpdrssYN    0.000                               0.000    0.000
##    .SIP19sitlongYN    0.000                               0.000    0.000
##    .SIP20nohjobsYN    0.000                               0.000    0.000
##    .SIP21irritblYN    0.000                               0.000    0.000
##    .SIP22slwstrsYN    0.000                               0.000    0.000
##    .SIP23staybedYN    0.000                               0.000    0.000
##    .SIP24allpainYN    0.000                               0.000    0.000
##     SIP               0.000                               0.000    0.000
## 
## Thresholds:
##                    Estimate  Std.Err  z-value  P(>|z|)   Std.lv  Std.all
##     SIP1styhmYN|t1    0.136    0.085    1.607    0.108    0.136    0.136
##     SIP2chngpsYN|1   -1.167    0.109  -10.718    0.000   -1.167   -1.167
##     SIP3slwwlkYN|1   -0.667    0.091   -7.294    0.000   -0.667   -0.667
##     SIP4notdoYN|t1    0.034    0.084    0.402    0.688    0.034    0.034
##     SIP5hndrlYN|t1    0.000    0.084    0.000    1.000    0.000    0.000
##     SIP6lidwnYN|t1   -0.263    0.085   -3.077    0.002   -0.263   -0.263
##     SIP7holdnYN|t1   -0.045    0.084   -0.536    0.592   -0.045   -0.045
##     SIP8thrsdYN|t1    0.406    0.087    4.674    0.000    0.406    0.406
##     SIP9slwdrsYN|1   -0.286    0.086   -3.344    0.001   -0.286   -0.286
##     SIP10shrtpYN|1   -0.286    0.086   -3.344    0.001   -0.286   -0.286
##     SIP11ntbndYN|1   -0.263    0.085   -3.077    0.002   -0.263   -0.263
##     SIP12dffchYN|1    0.079    0.084    0.937    0.349    0.079    0.079
##     SIP13dffbdYN|1   -0.239    0.085   -2.810    0.005   -0.239   -0.239
##     SIP14npptYN|t1    0.443    0.087    5.071    0.000    0.443    0.443
##     SIP15dffscYN|1   -0.125    0.085   -1.473    0.141   -0.125   -0.125
##     SIP16SshrtYN|1   -0.431    0.087   -4.939    0.000   -0.431   -0.431
##     SIP17bdslpYN|1   -0.967    0.100   -9.642    0.000   -0.967   -0.967
##     SIP18hlpdrYN|1    0.986    0.101    9.757    0.000    0.986    0.986
##     SIP19stlngYN|1    0.494    0.088    5.599    0.000    0.494    0.494
##     SIP20nhjbsYN|1   -0.770    0.094   -8.185    0.000   -0.770   -0.770
##     SIP21rrtblYN|1    0.068    0.084    0.804    0.422    0.068    0.068
##     SIP22slwstYN|1   -0.274    0.085   -3.210    0.001   -0.274   -0.274
##     SIP23stybdYN|1    1.313    0.117   11.237    0.000    1.313    1.313
##     SIP24llpnYN|t1   -0.967    0.100   -9.642    0.000   -0.967   -0.967
## 
## Variances:
##                    Estimate  Std.Err  z-value  P(>|z|)   Std.lv  Std.all
##    .SIP1stayhomeYN    0.463                               0.463    0.463
##    .SIP2changepsYN    0.493                               0.493    0.493
##    .SIP3slowwalkYN    0.452                               0.452    0.452
##    .SIP4notdoYN       0.640                               0.640    0.640
##    .SIP5handrailYN    0.481                               0.481    0.481
##    .SIP6liedownYN     0.841                               0.841    0.841
##    .SIP7holdonYN      0.352                               0.352    0.352
##    .SIP8othersdoYN    0.742                               0.742    0.742
##    .SIP9slowdrssYN    0.335                               0.335    0.335
##    .SIP10shortupYN    0.568                               0.568    0.568
##    .SIP11notbendYN    0.410                               0.410    0.410
##    .SIP12diffchrYN    0.315                               0.315    0.315
##    .SIP13diffbedYN    0.662                               0.662    0.662
##    .SIP14noappetYN    0.859                               0.859    0.859
##    .SIP15dffscksYN    0.443                               0.443    0.443
##    .SIP16SshrtwlYN    0.456                               0.456    0.456
##    .SIP17badslepYN    0.627                               0.627    0.627
##    .SIP18hlpdrssYN    0.281                               0.281    0.281
##    .SIP19sitlongYN    0.634                               0.634    0.634
##    .SIP20nohjobsYN    0.661                               0.661    0.661
##    .SIP21irritblYN    0.768                               0.768    0.768
##    .SIP22slwstrsYN    0.413                               0.413    0.413
##    .SIP23staybedYN    0.675                               0.675    0.675
##    .SIP24allpainYN    0.592                               0.592    0.592
##     SIP               0.537    0.078    6.898    0.000    1.000    1.000
## 
## Scales y*:
##                    Estimate  Std.Err  z-value  P(>|z|)   Std.lv  Std.all
##     SIP1stayhomeYN    1.000                               1.000    1.000
##     SIP2changepsYN    1.000                               1.000    1.000
##     SIP3slowwalkYN    1.000                               1.000    1.000
##     SIP4notdoYN       1.000                               1.000    1.000
##     SIP5handrailYN    1.000                               1.000    1.000
##     SIP6liedownYN     1.000                               1.000    1.000
##     SIP7holdonYN      1.000                               1.000    1.000
##     SIP8othersdoYN    1.000                               1.000    1.000
##     SIP9slowdrssYN    1.000                               1.000    1.000
##     SIP10shortupYN    1.000                               1.000    1.000
##     SIP11notbendYN    1.000                               1.000    1.000
##     SIP12diffchrYN    1.000                               1.000    1.000
##     SIP13diffbedYN    1.000                               1.000    1.000
##     SIP14noappetYN    1.000                               1.000    1.000
##     SIP15dffscksYN    1.000                               1.000    1.000
##     SIP16SshrtwlYN    1.000                               1.000    1.000
##     SIP17badslepYN    1.000                               1.000    1.000
##     SIP18hlpdrssYN    1.000                               1.000    1.000
##     SIP19sitlongYN    1.000                               1.000    1.000
##     SIP20nohjobsYN    1.000                               1.000    1.000
##     SIP21irritblYN    1.000                               1.000    1.000
##     SIP22slwstrsYN    1.000                               1.000    1.000
##     SIP23staybedYN    1.000                               1.000    1.000
##     SIP24allpainYN    1.000                               1.000    1.000
```

Figure 16: FA: 1-factor confirmatory factor analysis diagram 24 items

15-item SIP:

```
## lavaan (0.5-23.1097) converged normally after  23 iterations
## 
##   Number of observations                           222
## 
##   Estimator                                       DWLS      Robust
##   Minimum Function Test Statistic              143.932     186.897
##   Degrees of freedom                                90          90
##   P-value (Chi-square)                           0.000       0.000
##   Scaling correction factor                                  0.894
##   Shift parameter                                           25.887
##     for simple second-order correction (Mplus variant)
## 
## Model test baseline model:
## 
##   Minimum Function Test Statistic             3582.921    1950.455
##   Degrees of freedom                               105         105
##   P-value                                        0.000       0.000
## 
## User model versus baseline model:
## 
##   Comparative Fit Index (CFI)                    0.984       0.947
##   Tucker-Lewis Index (TLI)                       0.982       0.939
## 
##   Robust Comparative Fit Index (CFI)                            NA
##   Robust Tucker-Lewis Index (TLI)                               NA
## 
## Root Mean Square Error of Approximation:
## 
##   RMSEA                                          0.052       0.070
##   90 Percent Confidence Interval          0.036  0.068       0.056  0.084
##   P-value RMSEA <= 0.05                          0.399       0.012
## 
##   Robust RMSEA                                                  NA
##   90 Percent Confidence Interval                                NA     NA
## 
## Standardized Root Mean Square Residual:
## 
##   SRMR                                           0.102       0.102
## 
## Weighted Root Mean Square Residual:
## 
##   WRMR                                           1.095       1.095
## 
## Parameter Estimates:
## 
##   Information                                 Expected
##   Standard Errors                           Robust.sem
## 
## Latent Variables:
##                    Estimate  Std.Err  z-value  P(>|z|)   Std.lv  Std.all
##   SIP =~                                                                
##     SIP1stayhomeYN    1.000                               0.679    0.679
##     SIP2changepsYN    0.997    0.146    6.830    0.000    0.678    0.678
##     SIP3slowwalkYN    1.140    0.131    8.686    0.000    0.774    0.774
##     SIP5handrailYN    1.083    0.119    9.087    0.000    0.735    0.735
##     SIP9slowdrssYN    1.205    0.124    9.692    0.000    0.819    0.819
##     SIP10shortupYN    1.013    0.109    9.310    0.000    0.688    0.688
##     SIP11notbendYN    1.119    0.119    9.370    0.000    0.760    0.760
##     SIP12diffchrYN    1.166    0.118    9.841    0.000    0.792    0.792
##     SIP13diffbedYN    0.825    0.119    6.914    0.000    0.560    0.560
##     SIP15dffscksYN    1.117    0.124    9.031    0.000    0.758    0.758
##     SIP16SshrtwlYN    1.143    0.121    9.427    0.000    0.777    0.777
##     SIP19sitlongYN    0.885    0.115    7.709    0.000    0.601    0.601
##     SIP20nohjobsYN    0.843    0.127    6.620    0.000    0.573    0.573
##     SIP22slwstrsYN    1.196    0.124    9.672    0.000    0.813    0.813
##     SIP24allpainYN    0.919    0.137    6.729    0.000    0.624    0.624
## 
## Intercepts:
##                    Estimate  Std.Err  z-value  P(>|z|)   Std.lv  Std.all
##    .SIP1stayhomeYN    0.000                               0.000    0.000
##    .SIP2changepsYN    0.000                               0.000    0.000
##    .SIP3slowwalkYN    0.000                               0.000    0.000
##    .SIP5handrailYN    0.000                               0.000    0.000
##    .SIP9slowdrssYN    0.000                               0.000    0.000
##    .SIP10shortupYN    0.000                               0.000    0.000
##    .SIP11notbendYN    0.000                               0.000    0.000
##    .SIP12diffchrYN    0.000                               0.000    0.000
##    .SIP13diffbedYN    0.000                               0.000    0.000
##    .SIP15dffscksYN    0.000                               0.000    0.000
##    .SIP16SshrtwlYN    0.000                               0.000    0.000
##    .SIP19sitlongYN    0.000                               0.000    0.000
##    .SIP20nohjobsYN    0.000                               0.000    0.000
##    .SIP22slwstrsYN    0.000                               0.000    0.000
##    .SIP24allpainYN    0.000                               0.000    0.000
##     SIP               0.000                               0.000    0.000
## 
## Thresholds:
##                    Estimate  Std.Err  z-value  P(>|z|)   Std.lv  Std.all
##     SIP1styhmYN|t1    0.136    0.085    1.607    0.108    0.136    0.136
##     SIP2chngpsYN|1   -1.167    0.109  -10.718    0.000   -1.167   -1.167
##     SIP3slwwlkYN|1   -0.667    0.091   -7.294    0.000   -0.667   -0.667
##     SIP5hndrlYN|t1    0.000    0.084    0.000    1.000    0.000    0.000
##     SIP9slwdrsYN|1   -0.286    0.086   -3.344    0.001   -0.286   -0.286
##     SIP10shrtpYN|1   -0.286    0.086   -3.344    0.001   -0.286   -0.286
##     SIP11ntbndYN|1   -0.263    0.085   -3.077    0.002   -0.263   -0.263
##     SIP12dffchYN|1    0.079    0.084    0.937    0.349    0.079    0.079
##     SIP13dffbdYN|1   -0.239    0.085   -2.810    0.005   -0.239   -0.239
##     SIP15dffscYN|1   -0.125    0.085   -1.473    0.141   -0.125   -0.125
##     SIP16SshrtYN|1   -0.431    0.087   -4.939    0.000   -0.431   -0.431
##     SIP19stlngYN|1    0.494    0.088    5.599    0.000    0.494    0.494
##     SIP20nhjbsYN|1   -0.770    0.094   -8.185    0.000   -0.770   -0.770
##     SIP22slwstYN|1   -0.274    0.085   -3.210    0.001   -0.274   -0.274
##     SIP24llpnYN|t1   -0.967    0.100   -9.642    0.000   -0.967   -0.967
## 
## Variances:
##                    Estimate  Std.Err  z-value  P(>|z|)   Std.lv  Std.all
##    .SIP1stayhomeYN    0.539                               0.539    0.539
##    .SIP2changepsYN    0.541                               0.541    0.541
##    .SIP3slowwalkYN    0.401                               0.401    0.401
##    .SIP5handrailYN    0.459                               0.459    0.459
##    .SIP9slowdrssYN    0.330                               0.330    0.330
##    .SIP10shortupYN    0.527                               0.527    0.527
##    .SIP11notbendYN    0.422                               0.422    0.422
##    .SIP12diffchrYN    0.373                               0.373    0.373
##    .SIP13diffbedYN    0.686                               0.686    0.686
##    .SIP15dffscksYN    0.425                               0.425    0.425
##    .SIP16SshrtwlYN    0.397                               0.397    0.397
##    .SIP19sitlongYN    0.639                               0.639    0.639
##    .SIP20nohjobsYN    0.672                               0.672    0.672
##    .SIP22slwstrsYN    0.340                               0.340    0.340
##    .SIP24allpainYN    0.610                               0.610    0.610
##     SIP               0.461    0.082    5.606    0.000    1.000    1.000
## 
## Scales y*:
##                    Estimate  Std.Err  z-value  P(>|z|)   Std.lv  Std.all
##     SIP1stayhomeYN    1.000                               1.000    1.000
##     SIP2changepsYN    1.000                               1.000    1.000
##     SIP3slowwalkYN    1.000                               1.000    1.000
##     SIP5handrailYN    1.000                               1.000    1.000
##     SIP9slowdrssYN    1.000                               1.000    1.000
##     SIP10shortupYN    1.000                               1.000    1.000
##     SIP11notbendYN    1.000                               1.000    1.000
##     SIP12diffchrYN    1.000                               1.000    1.000
##     SIP13diffbedYN    1.000                               1.000    1.000
##     SIP15dffscksYN    1.000                               1.000    1.000
##     SIP16SshrtwlYN    1.000                               1.000    1.000
##     SIP19sitlongYN    1.000                               1.000    1.000
##     SIP20nohjobsYN    1.000                               1.000    1.000
##     SIP22slwstrsYN    1.000                               1.000    1.000
##     SIP24allpainYN    1.000                               1.000    1.000
```

Figure 17: FA: 1-factor confirmatory factor analysis diagram 15 items

## Interpretation

The parallel analysis propose 6 factors and 3 components, none being consistent with Step 2. The VSS and the item cluster analyses are however supportive of a single factor solution. The 1-factor 24-item SIP CFA has suboptimal fit (significant robust chi-square, GoF indices slightly below thresholds) and the 3 items which were unscalable in MSA show up here as the ones with the lowest loadings, yet higher than the .30 commonly used threshold. The 1-factor 15-item SIP had slightly better fit compared to the 24-item solution.

# Step 5: Classical Test Theory analysis

Several indices of scale reliability are displayed in Table 11 for each subscale: Cronbach’s alpha, Guttman’s lambda6, beta, omega (confidence intervals available in the script). Scale properties if item is dropped are reported for each subscale in separate tables (Table 12 to Table 13).

## Results

Table 14: CTT: Reliability indices all scales

| Scale | Calpha | G6 | Beta | Omega |
| --- | --- | --- | --- | --- |
| SIP24 | 0.88 [ 0.86 - 0.91 ] | 0.90 | 0.53 | 0.89 [ 0.88 - 0.91 ] |
| SIP15 | 0.86 [ 0.84 - 0.89 ] | 0.87 | 0.68 | 0.87 [ 0.85 - 0.88 ] |

Table 15: CTT: SIP24: Reliability if item dropped

|  | raw\_alpha | std.alpha | G6(smc) | average\_r | S/N | alpha se |
| --- | --- | --- | --- | --- | --- | --- |
| SIP1stayhomeYN | 0.88 | 0.88 | 0.9 | 0.24 | 7.08 | 0.01 |
| SIP2changeposYN | 0.88 | 0.88 | 0.9 | 0.24 | 7.38 | 0.01 |
| SIP3slowwalkYN | 0.88 | 0.88 | 0.9 | 0.24 | 7.21 | 0.01 |
| SIP4notdoYN | 0.88 | 0.88 | 0.9 | 0.24 | 7.29 | 0.01 |
| SIP5handrailYN | 0.88 | 0.88 | 0.9 | 0.24 | 7.18 | 0.01 |
| SIP6liedownYN | 0.88 | 0.88 | 0.9 | 0.25 | 7.58 | 0.01 |
| SIP7holdonYN | 0.88 | 0.88 | 0.9 | 0.24 | 7.10 | 0.01 |
| SIP8othersdoYN | 0.88 | 0.88 | 0.9 | 0.24 | 7.46 | 0.01 |
| SIP9slowdressYN | 0.88 | 0.88 | 0.9 | 0.23 | 7.02 | 0.01 |
| SIP10shortupYN | 0.88 | 0.88 | 0.9 | 0.24 | 7.26 | 0.01 |
| SIP11notbendYN | 0.88 | 0.88 | 0.9 | 0.24 | 7.08 | 0.01 |
| SIP12diffchairYN | 0.88 | 0.88 | 0.9 | 0.23 | 7.03 | 0.01 |
| SIP13diffbedYN | 0.88 | 0.88 | 0.9 | 0.24 | 7.36 | 0.01 |
| SIP14noappetYN | 0.89 | 0.88 | 0.9 | 0.25 | 7.64 | 0.01 |
| SIP15diffsocksYN | 0.88 | 0.88 | 0.9 | 0.24 | 7.15 | 0.01 |
| SIP16SshortwalkYN | 0.88 | 0.88 | 0.9 | 0.24 | 7.17 | 0.01 |
| SIP17badsleepYN | 0.88 | 0.88 | 0.9 | 0.24 | 7.43 | 0.01 |
| SIP18helpdressYN | 0.88 | 0.88 | 0.9 | 0.24 | 7.21 | 0.01 |
| SIP19sitlongYN | 0.88 | 0.88 | 0.9 | 0.24 | 7.37 | 0.01 |
| SIP20nohjobsYN | 0.88 | 0.88 | 0.9 | 0.24 | 7.41 | 0.01 |
| SIP21irritableYN | 0.88 | 0.88 | 0.9 | 0.24 | 7.45 | 0.01 |
| SIP22slowstairsYN | 0.88 | 0.88 | 0.9 | 0.24 | 7.10 | 0.01 |
| SIP23staybedYN | 0.88 | 0.88 | 0.9 | 0.25 | 7.58 | 0.01 |
| SIP24allpainYN | 0.88 | 0.88 | 0.9 | 0.24 | 7.38 | 0.01 |

Table 16: CTT: SIP15: Reliability if item dropped

|  | raw\_alpha | std.alpha | G6(smc) | average\_r | S/N | alpha se |
| --- | --- | --- | --- | --- | --- | --- |
| SIP1stayhomeYN | 0.86 | 0.85 | 0.87 | 0.29 | 5.83 | 0.01 |
| SIP2changeposYN | 0.86 | 0.86 | 0.87 | 0.30 | 6.10 | 0.01 |
| SIP3slowwalkYN | 0.85 | 0.85 | 0.86 | 0.29 | 5.76 | 0.01 |
| SIP5handrailYN | 0.85 | 0.85 | 0.86 | 0.29 | 5.75 | 0.01 |
| SIP9slowdressYN | 0.85 | 0.85 | 0.86 | 0.29 | 5.59 | 0.01 |
| SIP10shortupYN | 0.86 | 0.85 | 0.87 | 0.29 | 5.85 | 0.01 |
| SIP11notbendYN | 0.85 | 0.85 | 0.86 | 0.29 | 5.66 | 0.01 |
| SIP12diffchairYN | 0.85 | 0.85 | 0.86 | 0.29 | 5.63 | 0.01 |
| SIP13diffbedYN | 0.86 | 0.86 | 0.87 | 0.30 | 6.06 | 0.01 |
| SIP15diffsocksYN | 0.85 | 0.85 | 0.86 | 0.29 | 5.75 | 0.01 |
| SIP16SshortwalkYN | 0.85 | 0.85 | 0.86 | 0.29 | 5.67 | 0.01 |
| SIP19sitlongYN | 0.86 | 0.86 | 0.87 | 0.30 | 6.13 | 0.01 |
| SIP20nohjobsYN | 0.86 | 0.86 | 0.87 | 0.30 | 6.11 | 0.01 |
| SIP22slowstairsYN | 0.85 | 0.85 | 0.86 | 0.28 | 5.57 | 0.01 |
| SIP24allpainYN | 0.86 | 0.86 | 0.87 | 0.30 | 6.09 | 0.01 |

## Interpretation

Cronbach’s alpha and omega are above .80 for both versions of the questionnaire, while beta is improved for the 15-item version.

# Step 6: Total scores descriptives

Total scores are computed based on the previous decisions. Descriptive statistics are presented in table format. Distributions are shown graphically as histograms.

## Results

Table 17: Descriptive statistics total scores

|  | mean | sd | min | max | range | skew | kurtosis | se |
| --- | --- | --- | --- | --- | --- | --- | --- | --- |
| SIP24 | 13.05 | 5.75 | 0 | 24 | 24 | -0.16 | -0.89 | 0.39 |
| SIP15 | 9.21 | 4.09 | 0 | 15 | 15 | -0.44 | -0.91 | 0.27 |

Figure 20: Histogram total scores for subscales

Figure 21: Scatterplot and histograms: SIP24 vs SIP15, and SIP24 vs theta values based on SIP15

## Interpretation

Both the 24-item and 15-item SIP scores have acceptable distributions and summary statistics. Ceiling and floor effects are 1.35 and 0.9 % for the 24-item SIP and 5.86 and 1.8 % for the 14-item SIP, respectively. The two scores are highly correlated (Pearson’s r= 0.96).

# Sensitivity analyses

Table 18: Sensitivity analyses 24- versus 15-item SIP - Pearson’s correlations with related variables and regression models

|  | bipq1affect | bipq2long | bipq3control | bipq4treat | bipq5sympt | bipq6concern | bipq7underst | bipq8emot | mpqhowbad | SIP24 |
| --- | --- | --- | --- | --- | --- | --- | --- | --- | --- | --- |
| bipq2long | 0.43\*\*\* |  |  |  |  |  |  |  |  |  |
| bipq3control | -0.15\* | -0.18\*\* |  |  |  |  |  |  |  |  |
| bipq4treat | -0.15\* | -0.15\* | 0.42\*\*\* |  |  |  |  |  |  |  |
| bipq5sympt | 0.53\*\*\* | 0.29\*\*\* | -0.08 | -0.03 |  |  |  |  |  |  |
| bipq6concern | 0.57\*\*\* | 0.29\*\*\* | -0.27\*\*\* | -0.22\*\*\* | 0.41\*\*\* |  |  |  |  |  |
| bipq7underst | 0.09 | 0.06 | 0.26\*\*\* | 0.30\*\*\* | 0.03 | -0.08 |  |  |  |  |
| bipq8emot | 0.50\*\*\* | 0.25\*\*\* | -0.19\*\* | -0.14\* | 0.17\* | 0.60\*\*\* | -0.11 |  |  |  |
| mpqhowbad | 0.55\*\*\* | 0.30\*\*\* | -0.22\*\* | -0.15\* | 0.45\*\*\* | 0.40\*\*\* | 0.12# | 0.33\*\*\* |  |  |
| SIP24 | 0.64\*\*\* | 0.33\*\*\* | -0.14\* | -0.14\* | 0.46\*\*\* | 0.35\*\*\* | 0.04 | 0.35\*\*\* | 0.52\*\*\* |  |
| SIP15 | 0.61\*\*\* | 0.31\*\*\* | -0.11 | -0.12# | 0.44\*\*\* | 0.29\*\*\* | 0.05 | 0.26\*\*\* | 0.50\*\*\* | 0.96\*\*\* |

**Regression models, 15- and 24-item SIP**

|  | | |
|  | *Dependent variable:* | |
|  |  | |
|  | SIP15 | SIP24 |
|  | | |
| Intercept | -2.241 | -3.233 |
|  | (1.944) | (2.664) |
|  |  |  |
| Gender (male) | -0.013 | 0.320 |
|  | (0.482) | (0.660) |
|  |  |  |
| age | 0.024 | 0.007 |
|  | (0.020) | (0.028) |
|  |  |  |
| Education (low) | 0.805@ | 0.931 |
|  | (0.454) | (0.622) |
|  |  |  |
| Pain intensity - VAS | 0.027\*\* | 0.034\*\* |
|  | (0.008) | (0.011) |
|  |  |  |
| IP1 - consequences | 0.985\*\*\* | 1.285\*\*\* |
|  | (0.158) | (0.216) |
|  |  |  |
| IP2 - timeline | 0.038 | 0.112 |
|  | (0.157) | (0.215) |
|  |  |  |
| IP3 - personal control | -0.013 | -0.020 |
|  | (0.095) | (0.131) |
|  |  |  |
| IP4 - treatment control | -0.032 | -0.066 |
|  | (0.092) | (0.126) |
|  |  |  |
| IP5 - identity | 0.257@ | 0.423\* |
|  | (0.133) | (0.183) |
|  |  |  |
| IP6 - concern | -0.246\* | -0.326@ |
|  | (0.122) | (0.167) |
|  |  |  |
| IP7 - understanding | -0.028 | -0.037 |
|  | (0.087) | (0.119) |
|  |  |  |
| IP8 - emotional response | -0.016 | 0.201 |
|  | (0.117) | (0.161) |
|  |  |  |
|  | | |
| Observations | 222 | 222 |
| R2 | 0.452 | 0.480 |
| Adjusted R2 | 0.420 | 0.450 |
|  | | |
| *Note:* | @ p<.1; \* p<.05; \*\* p<.01; \*\*\* p<.001 | |

The results in Table 18 above present correlations with available variables in the dataset, and results of two multiple regression models predicting disability from background characteristics (gender, age, education level), pain intensity (as reported on a visual analogue scale (VAS)), and illness perceptions (as reported in the Brief Illness Perceptions Questionnaire - BIPQ). Results are largely similar, indicating that exclusion of the underperforming items did not lead to substantive changes. This increases the confidence in the results obtained with the 24-item SIP, and in the same time suggests that the 15-item version may be used in future studies in this sample to reduce patient burden.
